# Coarse-Grained Simulations of Polyrotaxane Hydrogels under Quiescent and Shear Conditions

**DOI:** 10.1021/acs.macromol.6c00624

**Published:** 2026-07-08

**Authors:** Cameron D. Smith, Wenlin Zhang

**Affiliations:** Department of Chemistry, 3728 Dartmouth College, Hanover, New Hampshire 03755, United States

## Abstract

Cyclodextrin-based polyrotaxanes (PR) form hydrogels in water through cyclodextrin (CD) aggregation and crystallization. These networks can break under shear flows, making them versatile platforms for extrusion-based 3D printing. To optimize the material properties of 3D-printed PR gels, a microscopic understanding of the structural evolution during 3D printing is necessary. Here, we employ coarse-grained (CG) simulations to reveal the PR assembly process at the molecular level. Our simulations reproduce the experimental crystal morphologies of PR at varying concentrations and chain lengths under quiescent conditions. Using nonequilibrium simulations, we show shear flow ruptures crystalline domain connectivity in PR gels and stacks the lamellae in the gradient direction, allowing the materials to flow during 3D printing. After the cessation of flow, the anisotropic crystal alignment and absence of available dangling PR diminish the intercrystal connectivity. The printed materials are therefore mechanically weaker than the pristine hydrogels, in agreement with experimental results. Nonetheless, by relating the microscopic structural evolution with viscoelastic properties of PR gels and solutions, we elucidate how flow conditions and sample composition affect the 3D printing performance of PR hydrogels.

## Graphical abstract

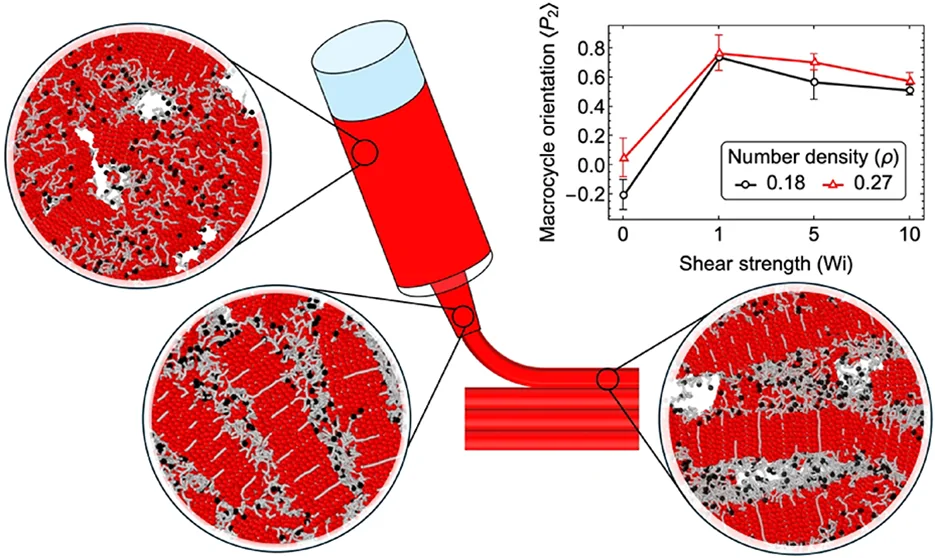

## Introduction

Extrusion-based 3D printing enables the fabrication of complex geometries with a high degree of customization, making it a valuable technique across fields such as biomedical engineering and soft robotics.^1–4^ The suitability of a hydrogel as an extrusion-based 3D printing ink is primarily governed by its rheological properties; the hydrogel must readily flow during deposition yet retain the shape of the scaffold thereafter.^1,2^ Polyrotaxanes (PR), composed of cyclodextrin (CD) threaded onto poly­(ethylene glycol) (PEG) chains, can form percolated hydrogel networks that exhibit shear thinning during printing and shape retention after deposition. This is due to the propensity for CD to form interchain hydrogen-bonded crystalline domains,^5^ which can be ruptured under stress and reform under quiescent conditions.^3,6^

The structure and connectivity of the PR crystalline domains are governed by the solution concentration, chain length, and macrocycle coverage. Consequently, the morphology of the crystalline domains can range from isolated nanosheets,^7^ which have found applications in biodegradable antifouling coatings and crystallization modifiers,^8,9^ to the percolated networks utilized as 3D printable inks.^10–12^ Understanding hydrogel formation in physically cross-linked polymers, however, remains a significant challenge due to their intrinsic structural heterogeneity and high water content.^13^

While the crystalline morphologies of PR nanosheets,^7^ single crystals,^10^ and brushes^14^ have been resolved experimentally, CD aggregation and network topology in PR hydrogels lack microscopic resolution.^10,15^ Prior experimental and theoretical studies have examined the assembly of individual PRs,^10,16,17^ and sol–gel transition of PR solutions,^18–20^ yet the presence of folded PRs in the crystalline domains has only recently been established.^7,14^ Additionally, PR hydrogels exhibit shear-induced rupture and alignment of crystalline domains,^21^ leading to pronounced shear-thinning and a decrease in elastic modulus after shear.^6,22–25^ However, the effect of the flow-induced structural evolution on the underlying material properties of the PR gel remains unclear. Addressing these gaps through a molecular-level understanding of the PR crystallization process will help in the rational design and optimization of 3D printable PR hydrogels.^3^

Molecular simulations can elucidate the assembly processes in polymer samples, including crystallization, at the microscopic level.^26–33^ Although atomistic simulations would be prohibitively expensive for applications to dilute hydrogel forming solutions, the spatiotemporal resolution of coarse-grained (CG) simulations are suitable for investigating the structural evolution in the PR hydrogels.^34^ In fact, CG simulations have readily been applied to study the molecular behaviors and properties of mechanically interlocked polymers,^35^ including polyrotaxanes in dilute solutions,^36–40^ slide-ring PR networks,^41,42^ and physically cross-linked elastomers.^43^ The effect of shear stress can also be integrated into simulations through nonequilibrium stochastic dynamics methods such as Langevin dynamics with an implicit flow field,^44,45^ or multiparticle collision dynamics (MPCD),^46–52^ which have been applied to polymer and colloidal solutions. These nonequilibrium simulations would help assess the structural evolution of PR solutions and hydrogels during the 3D printing processes.

In this work, we used CG simulations to elucidate the crystallization in PR solutions, which drives the formation of PR hydrogels under quiescent conditions. Our simulations reproduced the experimentally determined crystal morphology of CD-PEG-based PR solutions and networks. These networks exhibited shear thinning when exposed to fast shear flows and mimicked the disruption of the network and alignment of crystalline lamellae through the extrusion process. Low shear facilitates the merging of crystalline domains and formation of shear bands that stack in the gradient direction. Whereas higher shear rates mitigate band formation, yet still produce highly aligned lamellae.

After cessation of shear flow, we show that recrystallization alters both the crystalline morphology and network connectivity. Recovery following shear at all investigated rates and concentrations produces highly anisotropic crystalline orientation and reduced domain connectivity, in turn resulting in softer gels. We attribute this loss of connectivity to the limited availability of dangling polymer chains capable of bridging crystalline domains, which explains the decrease in modulus observed in experimental step-strain experiments of PR hydrogels. Nevertheless, when combined with chemical cross-linking, the layer-by-layer deposition of sheared PR samples can be used to create 3D printed soft scaffolds.^10,12^

## Methods

### Coarse-Grained Model

We sought to recover the experimentally determined crystalline structures in our CG simulations before exposing equilibrated PR solutions and gels to shear flow. Therefore, we adopted chain lengths (N) of 20–80 beads, where one bead is equal to one Kuhn segment (or three PEG monomers), corresponding to PEG_2.5k_–PEG_10k_.^17^ These chain lengths encompass several intermediate polymer lengths used by Uenuma et al.^7^ to determine the PR crystalline morphology, as well as the chain lengths of established 3D printable gels.^10–12^ Each PR had a coverage of 40%, a polymer bead to macrocycle ratio of 5:2, which falls in the middle of the experimental coverage of 30–60% used for 3D printing.^10,12^ All CG simulations were performed using LAMMPS (version 17 Apr 2024).^53–58^

Polymers and macrocycles were described by a generic bead–spring model (Figure 1), with all covalent bonds modeled as harmonic springs

1 $$U_{\mathrm{bond}}=K_{\mathrm{bond}}{\left(r_{\mathrm{ij}}-r_{0}\right)}^{2}$$

where *K*_bond_ is the spring constant, *r*_*ij*_ is the bond length between neighboring beads, and *r*_0_ is the equilibrium bond length. We set *r*_0_ to σ and *K*_bond_ to a constant of 300ϵ/σ ^2^, in which σ and ϵ are the reduced units of length and energy, respectively. Setting the mass *m* = 1 for all beads, we define the characteristic time in our simulations as τ = (*m*σ^2^/ϵ)^1/2^. The time step in all CG simulations was 0.01τ and simulations were performed at T* = 1ϵ*k*_B_^–1^ with a damping coefficient of 1τ.

**Figure 1:**
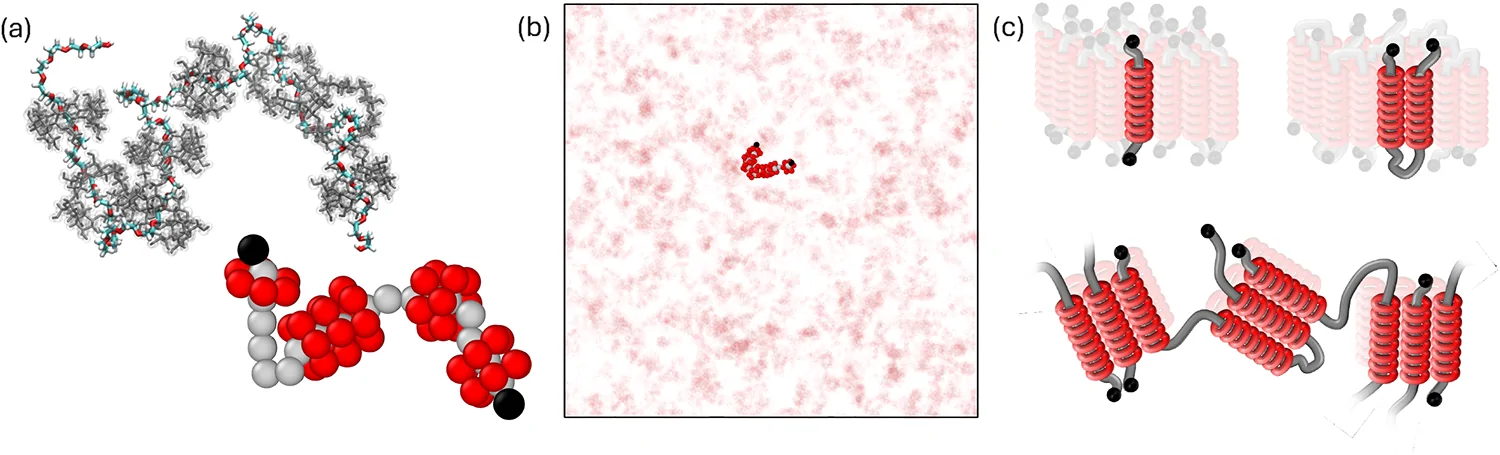
Coarse-grained model of PR. (a) Atomistic PEG_2.5k_ with eight α-cyclodextrin and the CG representation (N20). (b) CG simulations of a PR solution with one PR highlighted. (c) Crystalline PR morphologies clockwise starting from the upper left: stem lamellae, folded lamellae, and bridged clusters. All-atom and coarse-grained snapshots are visualized using VMD^59^ and OVITO,^60^ respectively.

Each chain contained mechanically interlocked macrocycles on the polymer, which maintained the ability to slide along the backbone. The polymer–polymer and polymer-macrocycle interactions were modeled as purely repulsive using the Weeks–Chandler–Anderson (WCA) potential.^61^

$$U_{WCA}=\{\begin{array}{l} 4\varepsilon_{\mathrm{ij}}[{\left(\frac{\sigma_{\mathrm{ij}}}{r_{\mathrm{ij}}}\right)}^{12}-{\left(\frac{\sigma_{\mathrm{ij}}}{r_{\mathrm{ij}}}\right)}^{6}+\frac{1}{4}],\mathrm{for}\;r_{\mathrm{ij}}<2^{1/6}\sigma_{\mathrm{ij}}\\ \;0,\mathrm{for}\;r_{\mathrm{ij}}\geq 2^{1/6}\sigma_{\mathrm{ij}} \end{array}$$ 2

where the indices *i*,*j* indicate different beads. We set σ_*ij*_ = 1.2σ for the interaction between the macrocycle and terminal bead on each chain to prevent the dethreading of the macrocycle, with σ_*ij*_ = σ and ϵ_*ij*_ = ϵ for all other interactions. Macrocycle-macrocycle interactions were also modeled with a WCA potential to generate random initial configurations. A shifted and truncated 12–6 Lennard–Jones (LJ) potential with a cutoff (*r*_*c*_) at 2.5σ was then applied to the ring beads to allow macrocycles to crystallize

$$U_{\mathrm{LJ}}=\{\begin{array}{l} 4\varepsilon_{M}\left[{\left(\frac{\sigma_{\mathrm{ij}}}{r_{\mathrm{ij}}}\right)}^{12}-{\left(\frac{\sigma_{\mathrm{ij}}}{r_{\mathrm{ij}}}\right)}^{6}-{\left(\frac{\sigma_{\mathrm{ij}}}{r_{c}}\right)}^{12}+{\left(\frac{\sigma_{\mathrm{ij}}}{r_{c}}\right)}^{6}\right],\mathrm{for}\;r_{\mathrm{ij}}<r_{c}\\ 0,\mathrm{for}\;r_{\mathrm{ij}}\geq r_{c} \end{array}$$ 3

where σ_*ij*_ = σ and ϵ_*M*_ is the macrocycle–macrocycle attraction. We set ϵ_*M*_ = 0.6ϵ to recover the experimentally determined crystalline morphology (Figure S1).

Our macrocycles consisted of six beads bonded by the harmonic potential *U*_bond_. To retain the shape of a hexagonal ring, we applied a harmonic angle potential

4 $$U_{\mathrm{angle}}=K_{\mathrm{angle}}{\left(\theta_{\mathrm{ijk}}-\theta_{0}\right)}^{2}$$

in which *K*_angle_ is the bending constant, θ_*ijk*_ is the angle between three successive macrocycle beads, and θ_0_ is the equilibrium angle. We set *K*_angle_ to 100ϵ/rad^2^ and θ_0_ to 2 π/3. A dihedral potential was used to preserve the planarity of the macrocycle through

5 $$U_{\mathrm{dihedral}}=\sum_{n=1,5}A_{n}\cos{\left(\phi_{\mathrm{ijkl}}\right)}^{\left(n-1\right)}$$

where ϕ_*ijkl*_ is the dihedral angle between four successive macrocycle beads, *A*_1_ = 20ϵ and *A*_3_ = −20ϵ, and *A*_2_ = *A*_4_ = *A*_5_ = 0.

A more realistic representation of PRs would incorporate the anisotropic nature of the CD interactions, similar to previous coarse-grained models.^62,63^ However, we restrict ourselves to the simplified isotropic LJ potential to enable the large system size and time scale required to model hydrogel formation. Our method is motivated by the work of Yasuda et al.,^40^ which showed that a six-bead macrocycle with a WCA potential exhibits diffusive behavior along a bead–spring polymer that is consistent with the experimentally observed 1-dimensional diffusion of αCD on PEG axles.^17,64^ We also find that our simple representation captures the correct crystal packing and domain morphology of CD in PR, thereby justifying the use of our model (see Results and Discussion section).

### Infinite PR Crystals

For comparison with our CG model, we employed all-atom (AA) MD simulations using GROMACS (version 2021.2)^65^ to characterize the structure of an infinite PR crystal slab with the CHARMM36 force field.^66^ The axial PEG polymer was built and parametrized using CHARMM-GUI,^67–70^ and the force field parameters for α-cyclodextrin (αCD) were obtained through CGenFF.^71–73^ A 1.2 nm cutoff distance was applied to all short-range interactions and the particle mesh Ewald method computed the long-range electrostatic interactions.^74^ All covalent bonds involving hydrogen were constrained using LINCS^75^ and a time-step of 2 fs was used for all AA simulations.

We created a PEG chain of 16 monomers and placed eight αCD head-to-head and tail-to-tail along the backbone, giving a 2:1 ratio of monomer to αCD and αCD orientations which match the single crystal data.^10^ A bond was introduced between the terminal oxygen and carbon on the PEG, at the sacrifice of two hydrogen atoms, to create a PR with a periodically infinite PEG axle. A total of 36 PR were then packed in a hexagonal lattice (Figure 2a) in a box of side lengths 6.7 × 7.7 × 6.0 nm, with the PR axle orientated along the *z*-axis. No water molecules were introduced to the crystal as they are only found in the form of hydrogen bonded bridges between the terminal αCD,^10^ of which there are none in the periodic crystal we created.

**Figure 2:**
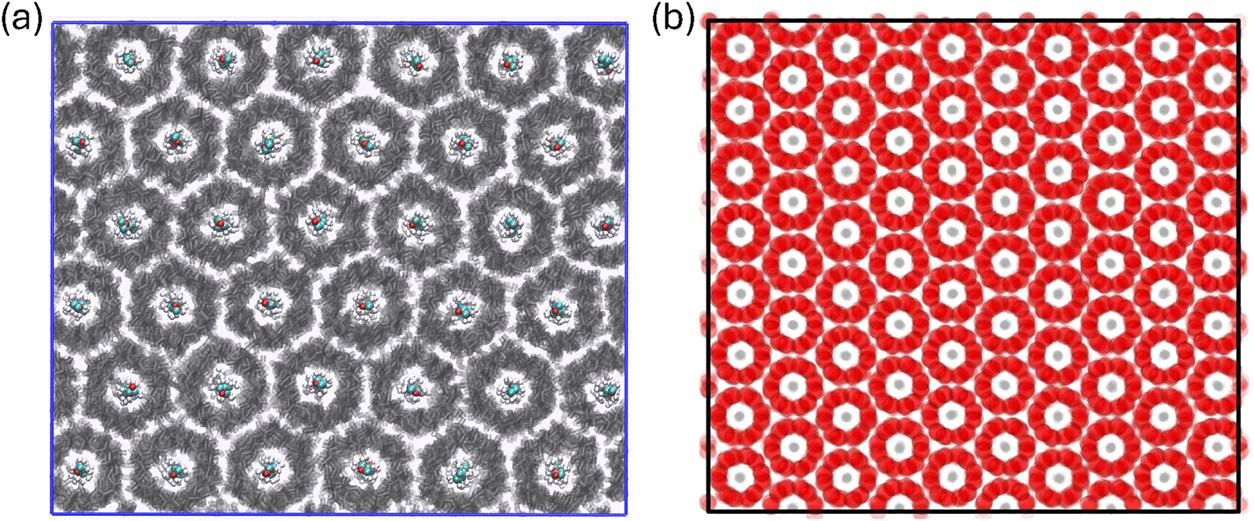
PR crystal structures of: (a) AA, and (b) CG simulations.

The crystal was initially equilibrated under NVT conditions at 300 K with the stochastic velocity rescale thermostat (v-rescale)^76^ for 1 ns. This was followed by a 10 ns NPT equilibration at 300 K with v-rescale and 1 bar using anisotropic pressure coupling with the Berendsen barostat.^77^ The system then underwent a production run of 25 ns at 300 K and 1 bar using v-rescale and semi-isotropic pressure coupling with the stochastic cell rescale barostat (c-rescale),^78^ respectively. This allowed the *x*/*y* plane (radial direction) and *z*-axis (axial direction) to fluctuate independently.

We also created a CG representation of the infinite PR crystal in which a PR chain consisted of 20 backbone beads and 20 threaded macrocycles. A total of 80 PR were then packed in a hexagonal lattice (Figure 2b) in a box of side lengths 25.0 × 21.7 × 20.0σ, with the PR axle orientated along the *z*-axis. A bond across the periodic boundary was introduced between the terminal beads of the axle polymers to create periodic PRs.

The CG crystal was equilibrated under NVT conditions at a temperature of T* = 1ϵ*k*_B_^–1^ for 100τ, followed by a 10^3^ τ NPT equilibration at T* = 1ϵ*k*_B_^–1^ and P* = 0ϵσ^–3^ with anisotropic pressure coupling using the Nosé-Hoover thermostat and barostat.^79^ The system then underwent a production run of 2.5 × 10^3^τ at T* = 1ϵ*k*_B_^–1^ and P* = 0ϵσ^–3^ with Nosé-Hoover thermostat and semi-isotropic pressure coupling, allowing the *x*/*y* plane and *z*-axis to fluctuate independently, matching the AA treatment.

### Crystallization

We created CG systems with implicit solvent and bead number densities for PR (the total number of polymer backbone and ring beads) ranging from approximately 0.09–0.27σ^–3^ to encompass the formation of isolated nanosheets and 3D-printable hydrogels. To assist the data visualization and the subsequent discussion of results, we use the dimensionless number density ρ (normalized using the unit volume σ^3^) and round the number densities across each chain length to group them into five representative values: 0.09, 0.13, 0.18, 0.23, and 0.27. The details of our quiescent simulation systems, including polymer lengths, number of PRs, box sizes, and ρ, are listed in Table 1. These parameters allow us to simulate PR crystallization in quiescent conditions. However, to fully capture the shear-induced structural changes, we later expand the simulation size to avoid finite size effects (see Results and Discussion section).

**Table 1:** Chain Length, Box Length, and Number Density (ρ) of Polyrotaxane Systems under Quiescent Conditions

Chain length	Number of PRs	Box length (σ)	ρ
20	648	79	0.089
		69	0.134
		62	0.185
		58	0.226
		55	0.265
40	324	79	0.089
		69	0.134
		62	0.185
		58	0.226
		55	0.265
60	324	90	0.091
		79	0.134
		72	0.177
		66	0.230
		62	0.277
80	324	99	0.091
		79	0.179
		69	0.268

We simulated the crystallization in quiescent conditions using Langevin dynamics. Each system was initially randomized for 20τ_*R*_ with a WCA potential for all interactions, where τ_*R*_ is the relaxation time of the end-to-end vector autocorrelation function in the most concentrated solution (ρ ≈ 0.27, Figure S2). Each density and chain length was run in triplicate, resulting in a total of 54 crystallization simulations. The attractive LJ potential was then enabled between macrocycles to promote the aggregation of rings along polymer backbones and the systems were allowed to crystallize for a maximum of 800τ_*R*_, until the crystallinity and number of crystalline domains plateaued (Figures S4–S5).

### Crystalline Domain Analyses

We determined crystalline order using the local orientational order (*S*) of macrocycle aggregates. To do so, we computed the center of mass and the normal vector (**t**) to the face of each macrocycle, from which a neighbor list for each macrocycle was created using a cylindrical cutoff scheme. The cutoff threshold was a center of mass displacement of 1.25σ normal to the macrocycle face and 3.0σ in the radial direction. The CG macrocycles packed into a hexagonal lattice when forming crystals, which matched experimental single-crystal data,^10^ giving a total of 20 neighboring sites within the cutoff distances per macrocycle.

To search for crystalline macrocycles, we only focused on rings with a neighbor site occupancy of 25% or greater. We take the largest eigenvalue (λ) of the local nematic tensor (*Q*)­

6 $$Q=\left\langle t⊗t\right\rangle -\frac{1}{3}I$$

where ⟨···⟩ denotes averaging over all the orientations of the neighboring macrocycles, and *I* is the identity matrix. The value of local orientational order *S* = 1.5λ can range from 0 (isotropic) to 1, which indicates perfect uniaxial alignment. We consider a macrocycle with a local order parameter *S* ≥ 0.9 as crystalline (see Supporting Information), and group crystalline macrocycles based on their neighbor list to identify the crystalline domains.

To quantify the orientational order in shear flows, we use the second-order Legendre polynomial (*P*_2_)­

7 $$P_{2}=\frac{3\cos{\left(\beta \right)}^{2}-1}{2}$$

where β is the deflection angle between **t** and the gradient direction (**n**). The value of *P*_2_ ranges from −0.5 to 1, indicating alignment perpendicular (−0.5) or parallel (1) to **n**.

### Shear Flow

Shear flow was introduced into samples with different PR concentrations to model hydrogel extrusion in 3D printing through the introduction of an external flow field. This method enforced the velocity profile of a background solvent on the PR system without the need to introduce an explicit solvent. The implicit flow field with a shear rate (γ̇) was applied through the introduction of a velocity tensor (Γ̂) into the Langevin dynamics equation^44^

8 $$ẍm_{\mathrm{LJ}}=F_{p}+F_{b}-\zeta \left(ẋ-x\cdot \mathrm{\Gamma ̂}\right)$$

where **x** is the particle position, *F*_*p*_ is the conservative force due to neighboring particles, *F*_*b*_ is a rapidly varying, zero-mean random force imposed by the implicit solvent molecules, and ζ is the damping coefficient of the implicit solvent. Here, Γ̂ takes the form

9 $$\mathrm{\Gamma ̂}=\left(\begin{array}{lll} 0 & \mathrm{\gamma ̇} & 0\\ 0 & 0 & 0\\ 0 & 0 & 0 \end{array}\right)$$

This resulted in *x*, *y*, and *z* being the flow, gradient, and vorticity directions, respectively. The direct implementation of the implicit flow field in Langevin dynamics does not account for hydrodynamic interactions (HI).^44^

Shear rates were chosen to match Weissenberg numbers (*Wi*), where *Wi* = γ̇τ_*R*_ and τ_*R*_ is the end-to-end vector relaxation time of uncrystallized PR chains. We chose *Wi* of 1, 5, and 10 to account for the influence of different printing speeds, which can affect the mechanical properties of the resultant gel.^80,81^ Each sample was sheared for a total strain (γ) of at least 150. We monitored the number of crystalline domains to ensure a steady state had been reached during the shearing process. The flow field was then removed and the sheared systems were re-equilibrated under quiescent conditions using a Langevin integrator for a total of 100τ_*R*_.

## Results and Discussion

### PR Samples under Quiescent Conditions

We first compared the crystalline structures in our AA and CG simulations. We determined the center of mass (COM) of each PEG axle in the AA crystalline slab and ascertained the radial distribution function (*g*(*r*)) in the plane perpendicular to the PEG alignment. The resultant distribution gave the packing characteristics of the PR crystal with nearest neighbor peak at 1.40 nm, followed by a second layer peak at 2.42 nm, and a third layer (an integer multiple of the nearest neighbor) at 2.79 nm (Figure 3). The second peak is in good agreement with experimentally determined channel spacing of 2.38 nm for the second PR layer.^10^ We performed the same analysis on the CG model by taking the COM of the axial polymer. By converting the reduced length unit (σ) to real units through *r* = σ × 0.5 nm, the CG *g*(*r*) matches the atomistic radial distribution function. Thus, our CG model accurately captures the hexagonally packed domains observed in crystalline PR.

**Figure 3:**
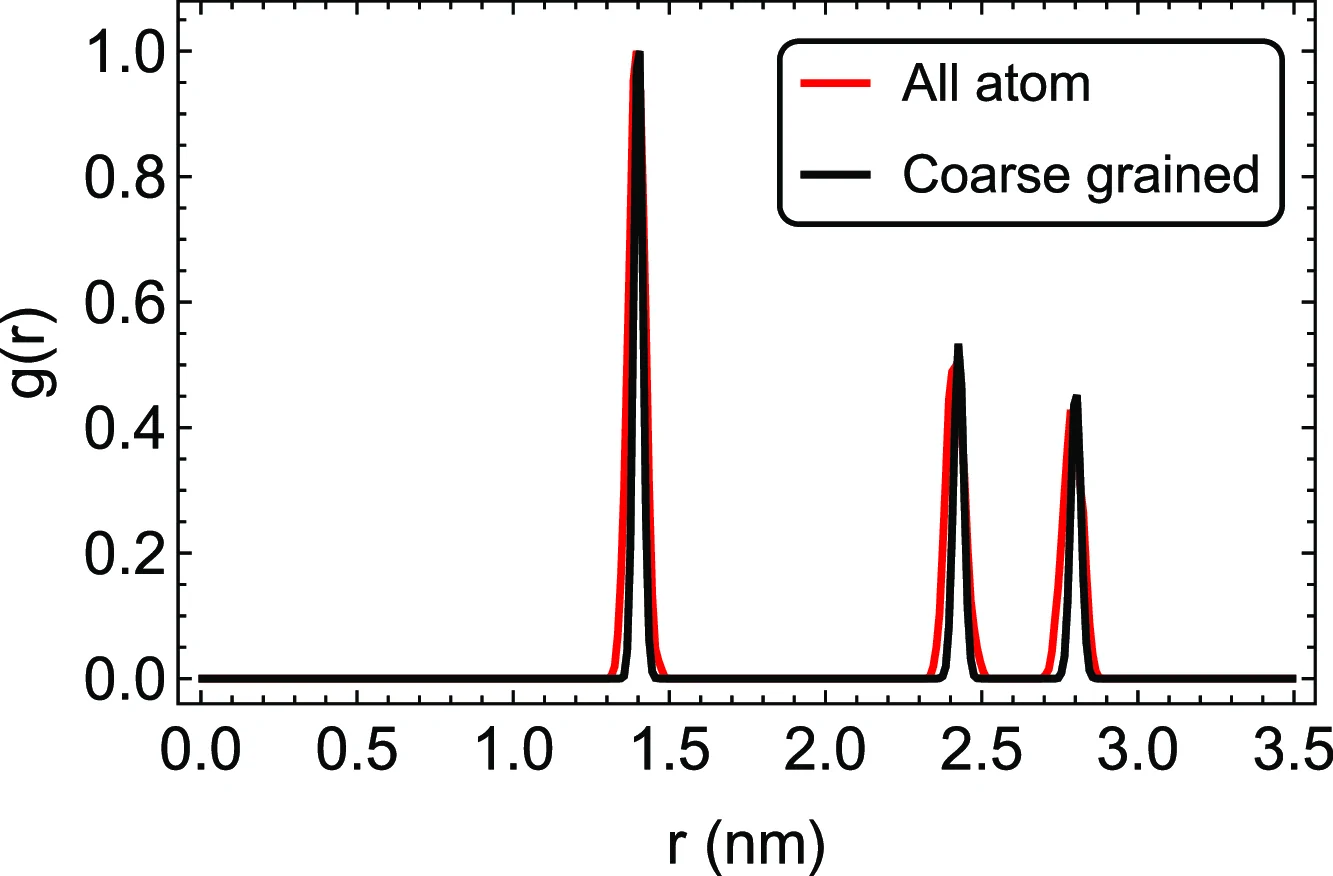
Pair-correlation *g*(*r*) in the radial direction of the polymer axle, using the COM of the PEG and CG backbone beads.

After we validated that our CG model yields the correct crystal packing, we sought to recover the PR crystallization behavior in PR systems. We identified the crystalline macrocycles in our subcooled PR systems, after “quenching” them by turning on the macrocycle attractive interactions, using the local nematic order parameter (*S*) and monitored the crystallinity (fraction of crystalline macrocycles) of our system. We also determined the crystal morphology by quantifying the composition of stem PR and folded PR (Figure 1c) present in the crystalline domains. Finally, we assessed the intercrystal connectivity by identifying polymers that bridged two or more domains.

Concentration and chain length had minimal influence on the final crystal fraction of the PR systems, and all systems reached a crystallinity of greater than 0.9 (Figure 4a). The composition of stem PR and folded PR was dramatically influenced by the chain length and slightly sensitive to the concentration. The crystalline domains of the *N*20 chains were almost exclusively comprised of single stem PR chains, with a folded fraction ranging from 0.07–0.15 in the low to high concentrations, respectively (Figure 4b). The high stem composition, along with the absence of any polymers bridging crystalline domains (Figure 5a), confirmed that these short chains have successfully formed the stem lamellae found experimentally.^7^

**Figure 4:**
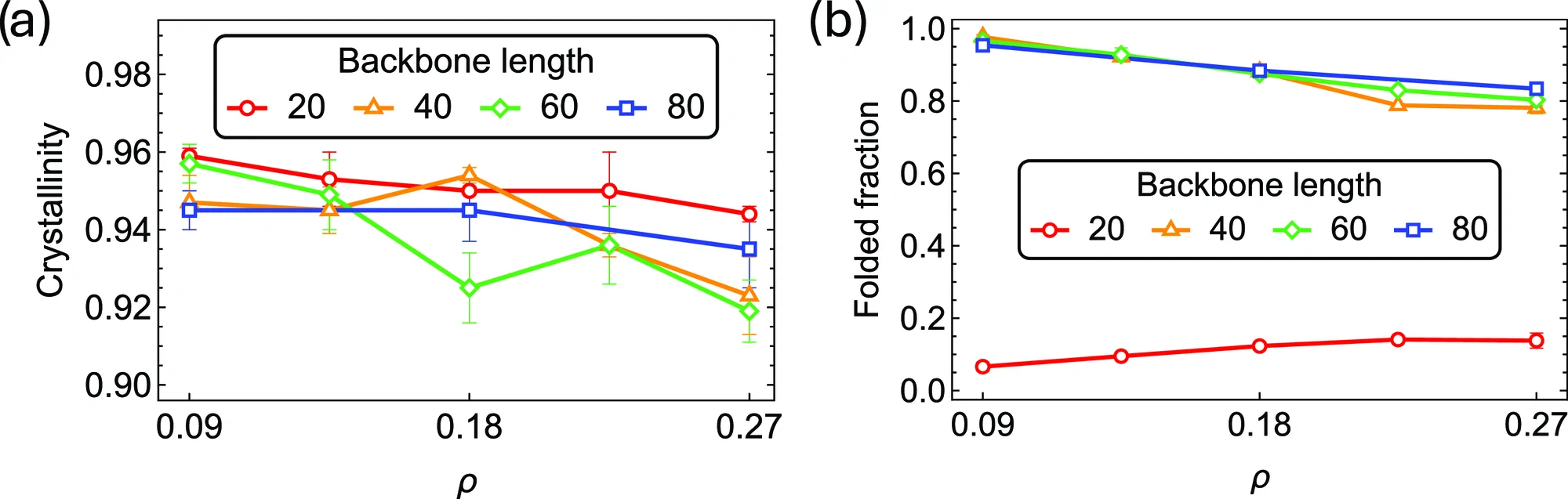
Concentration and chain length effect on: (a) crystallinity, and (b) fraction of crystalline macrocycles present in a folded conformation. Error bars represent the standard error from the separate simulations.

**Figure 5:**
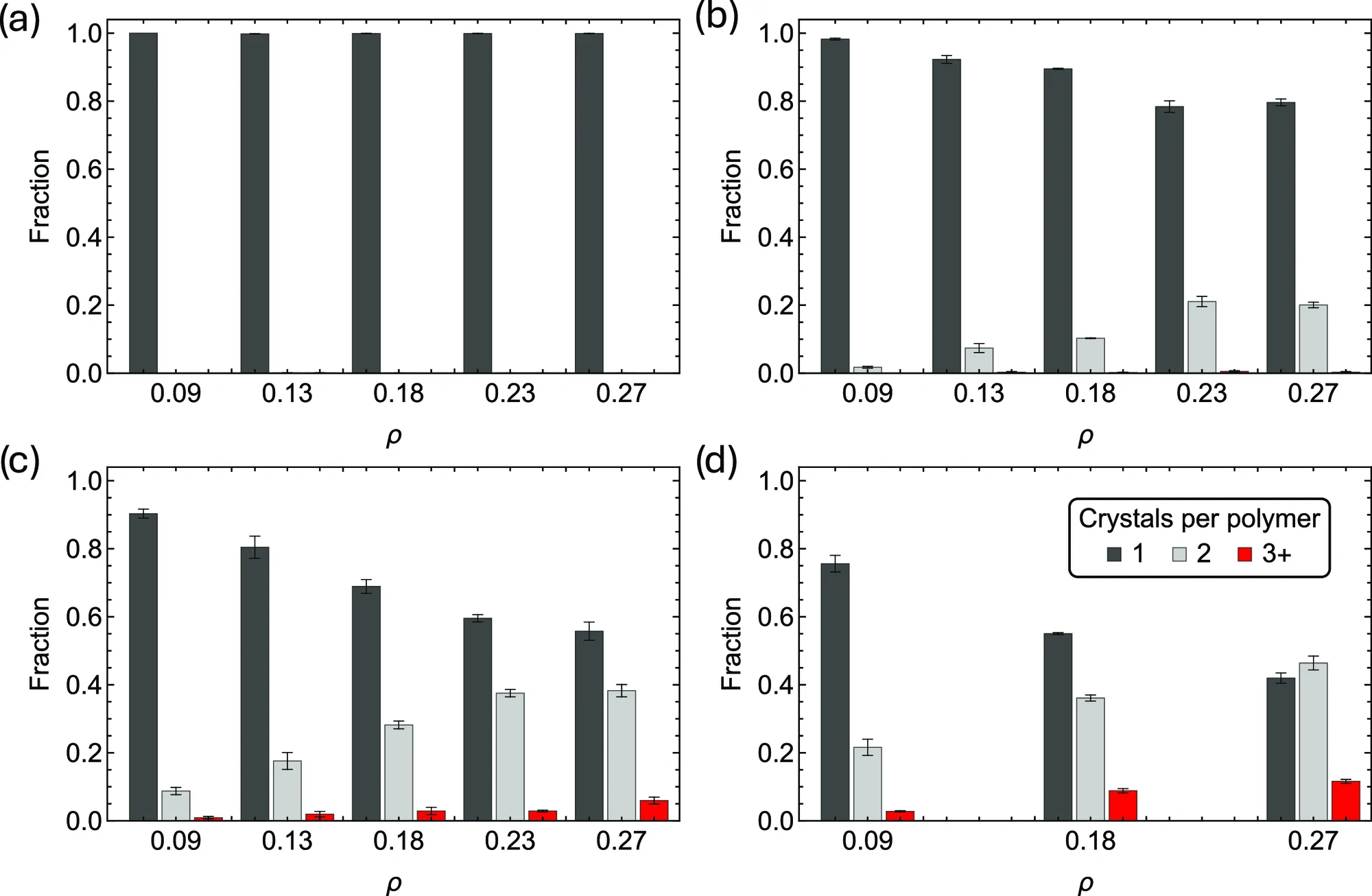
Concentration effect on the number of crystalline domains a single polymer chain spans. Chain lengths: (a) 20, (b) 40, (c) 60, and (d) 80.

The longer PR all formed crystalline domains with high fractions of folded PR (0.8–1.0), which were independent of the chain length but decreased as concentration increased (Figure 4b). Chain lengths of *N*40 exhibited some intercrystal connectivity, with the fraction of polymers bridging two crystalline domains increasing from 0.0 to 0.2 for the dilute to the concentrated systems (Figure 5b). As expected, longer chain lengths and higher concentrations resulted in a higher connectivity with some polymers bridging three or more domains (Figure 5c,d).

We combined the analyses of crystal composition and domain connectivity to construct a morphology diagram showing how concentration and chain length influence crystal morphology (Figure 6). We chose a cutoff of 0.1 for the fraction of polymers that span two or more domains to determine the threshold at which we transition from isolated sheets to “bridged clusters”. A value of 0.2 was chosen for the threshold folded fraction at which we transition from “stem lamellae” to “folded lamellae”. Any threshold value less than 0.8 for folded fraction yields the same morphological diagram. The PR crystal morphology determined by Uenuma et al.^7^ observed the formation of stem lamellae, folded lamellae, and bridged clusters at ρ ≈ 0.13 and chain lengths of 20, 40, and 60 beads, respectively, matching our morphology diagram. Our model can reliably capture the crystalline morphologies in PR systems under quiescent conditions.

**Figure 6:**
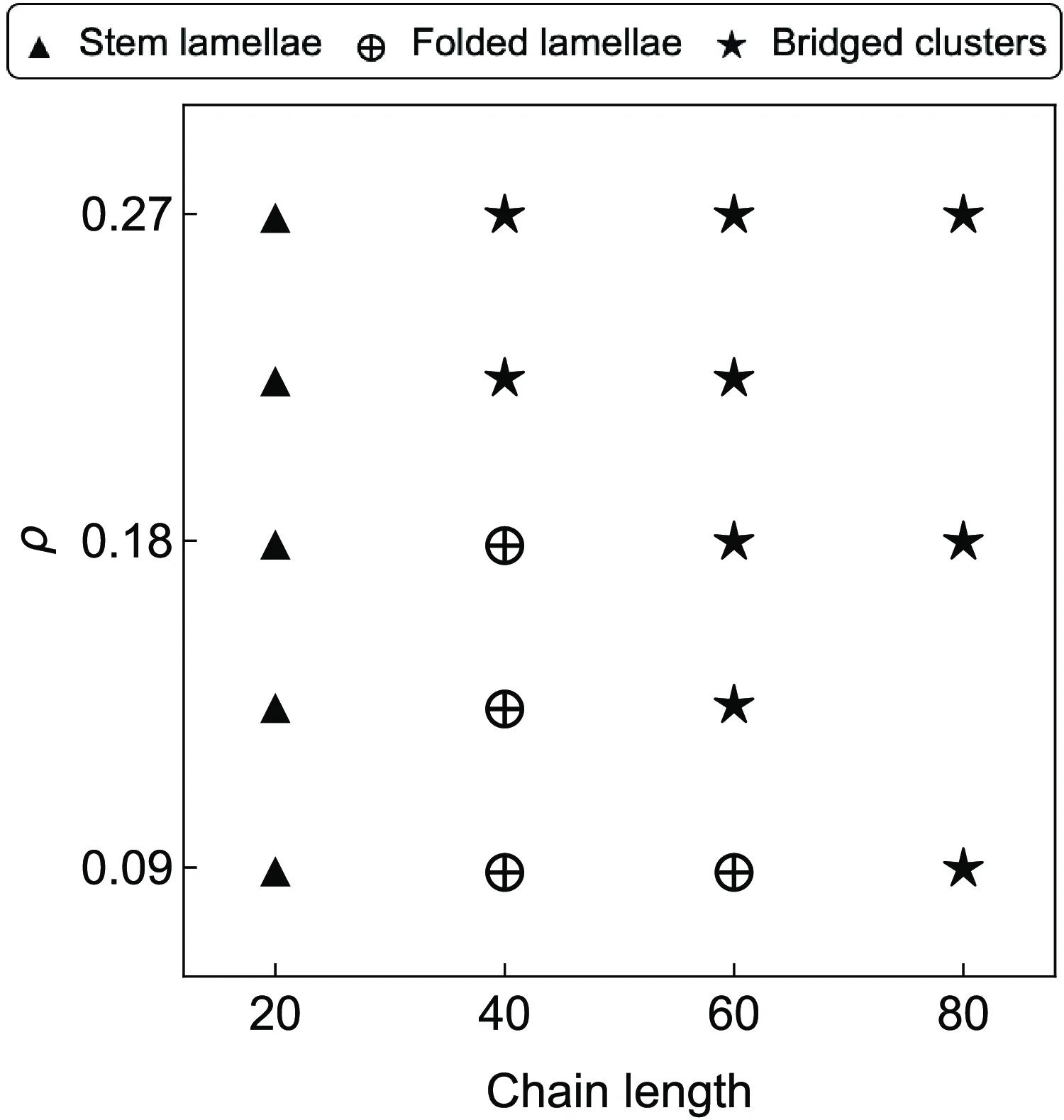
Crystalline domain morphology diagram of the systems tested.

In fact, crystalline clusters can percolate through space, enabling sufficiently concentrated PR systems to undergo a sol–gel transition and form an interconnected network. To see this, we checked if a crystalline cluster in our simulations is connected to itself through a periodic boundary.^82–84^ Space-spanning crystalline percolation is evaluated by determining whether an individual crystalline domain is connected to its own periodic image (Figure 7a). Bridging percolation is quantified using a graph representation, where crystalline domains are defined as nodes and bridging polymer chains as edges. Percolation is identified by detecting nodes that are connected to their periodic image along the *x*, *y*, or *z* directions (Figure 7b).

**Figure 7:**
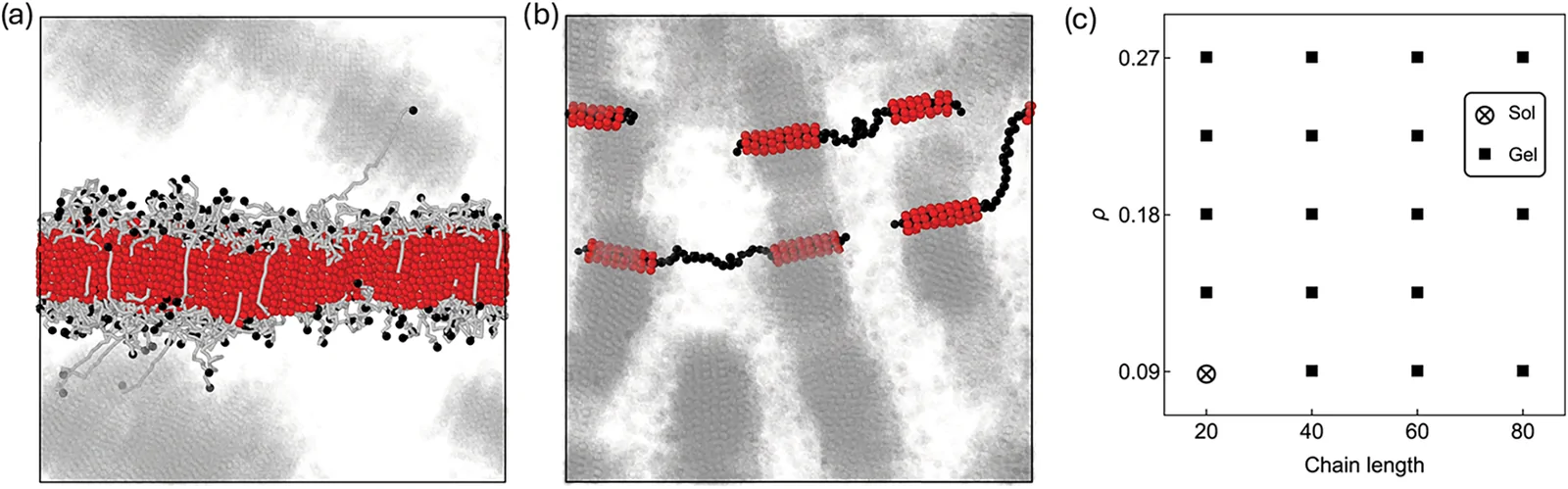
Snapshots of (a) space-spanning periodic crystal, and (b) bridging polymers connecting crystalline domains. (c) Sol–gel diagram of crystalline domain connectivity forming a percolated network.

We performed percolation analysis on the equilibrated PR systems at each density and chain length. Figure 7c shows that in dilute systems of short chains (N20), the lack of intercrystalline polymer bridges prevented any formation of a percolated gel. However, the concentrated N20 systems (ρ ≥ 0.13) achieved percolation through the formation of space-spanning periodic crystals. All systems with chain lengths of N40 and longer formed percolated gels (Figure 7c), with a notable result being that the N40 at ρ ≈ 0.27 formed a percolated gel, as this corresponds to PR systems which have successfully been utilized as functional 3D printable hydrogels.^10–12^

### PR Networks in Shear Flows

We next examined how PR networks respond to shear stress by introducing a flow field into the Langevin dynamics, resulting in the *x*, *y*, and *z* being the flow, gradient, and vorticity, respectively (Figure 8a). This provides a model for the shear conditions encountered during extrusion-based 3D hydrogel printing. To mimic the 3D-printable hydrogels, we simulate PR with length *N*40 at two concentrations ρ ≈ 0.18 and ρ ≈ 0.27. At high shear rates (*Wi* = 10), the enforced flow profile resulted in the formation of periodic column-like structures aligned in the vorticity direction (Figure 8b). Although these so-called “log-rolling” structures are a known confinement-induced phenomenon,^85^ they are unphysical in bulk solutions where the column-like structures are expected to align along the flow direction.^86^

**Figure 8:**
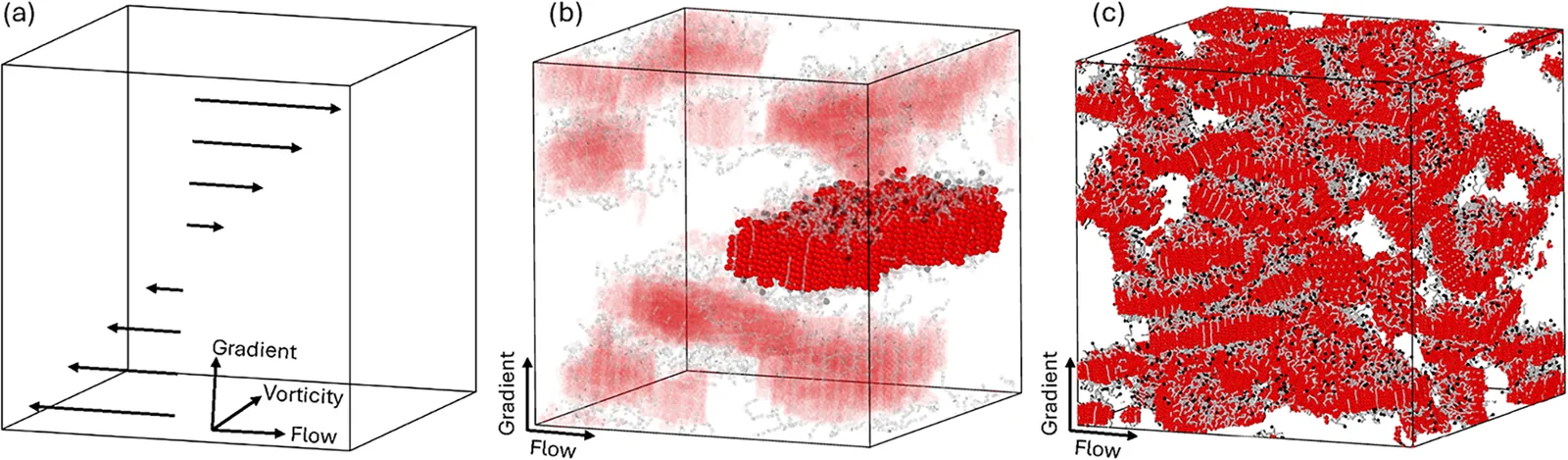
Nonequilibrium CG simulation of shearing PR systems. (a) A representation of the flow profile during shear. Snapshots at ρ = 0.18 and *Wi* = 10 of: (b) the “log-rolling” (vorticity aligned) crystallites formed due to the small box size, and (c) finite crystalline domains formed after increasing the system.

The rolling periodic structures did not result from the lack of hydrodynamic interactions (HI) in our Langevin dynamics simulations. To see this, we performed multiparticle collision dynamics (MPCD)^46,47,87^ simulations using HOOMD-blue (version 6.1.1).^88^ The Müller–Plathe method employed to generate shear flow in the MPCD simulations produces a triangular velocity profile,^89,90^ which differs from the Langevin dynamics method. This velocity profile can introduce artifacts in flow-induced structural rearrangement when large mesoscale structures span the two velocity regions.^52^ The possible formation of artifacts would need to be addressed if we used the MPCD simulations to study the sheared networks. Nonetheless, vorticity-aligned crystalline domains also formed in the presence of HI in the MPCD simulations, indicating that the incorporation of HI did not mitigate the formation of the rolling logs (see Supporting Information for further discussion).

We established that the “log-rolling” crystals were due to the finite-size effects of the box. As the crystalline domains tumble in the vorticity plane of the smaller box, the anisotropic “rods” tend to tilt toward the vorticity direction to avoid collisions with other tumbling crystals. If the box dimension is not large enough, tilted crystals can merge with their periodic image to establish a periodic crystal, which prevents realignment in the flow direction. Increasing the box size prevented the formation of these periodic vorticity-aligned columns (Figure 8c). Therefore, we expanded the system in all directions by replicating the equilibrated box in the *x*, *y*, and *z* periodic boundaries. This resulted in cubic boxes of side length 110σ and 124σ for the ρ ≈ 0.27 and 0.18, respectively, with each system containing 2592 PRs and 352,512 beads.

Nonetheless, shear flows can indeed rearrange the crystalline domains in the PR hydrogels. Exposing the hydrogels to shear flow at a shear of *Wi* = 1 resulted in large crystals forming perpendicular to the gradient direction in the dilute and concentrated samples, with multiple stable 2-dimensional periodic crystals forming at both densities (Figures 9a and S15 and S17). Our simulations are consistent with experimentally observed crystal stacking in the gradient direction in the sheared samples of polyrotaxanes composed of PEO–PPO-PEO block copolymers and β-cyclodextrin.^21^ The concentrated system also formed periodic lamellae under shear flows at *Wi* = 5; however, fast flows mitigated the formation of 2D shear bands in the dilute system. Even in the absence of shear banding, the crystalline domains retained a preferential orientation perpendicular to the gradient direction. No system sheared at *Wi* = 10 formed 2D periodic crystals, yet the small crystalline domains still retained an anisotropic orientation under flow (Figures 9b and S16 and S18).

**Figure 9:**
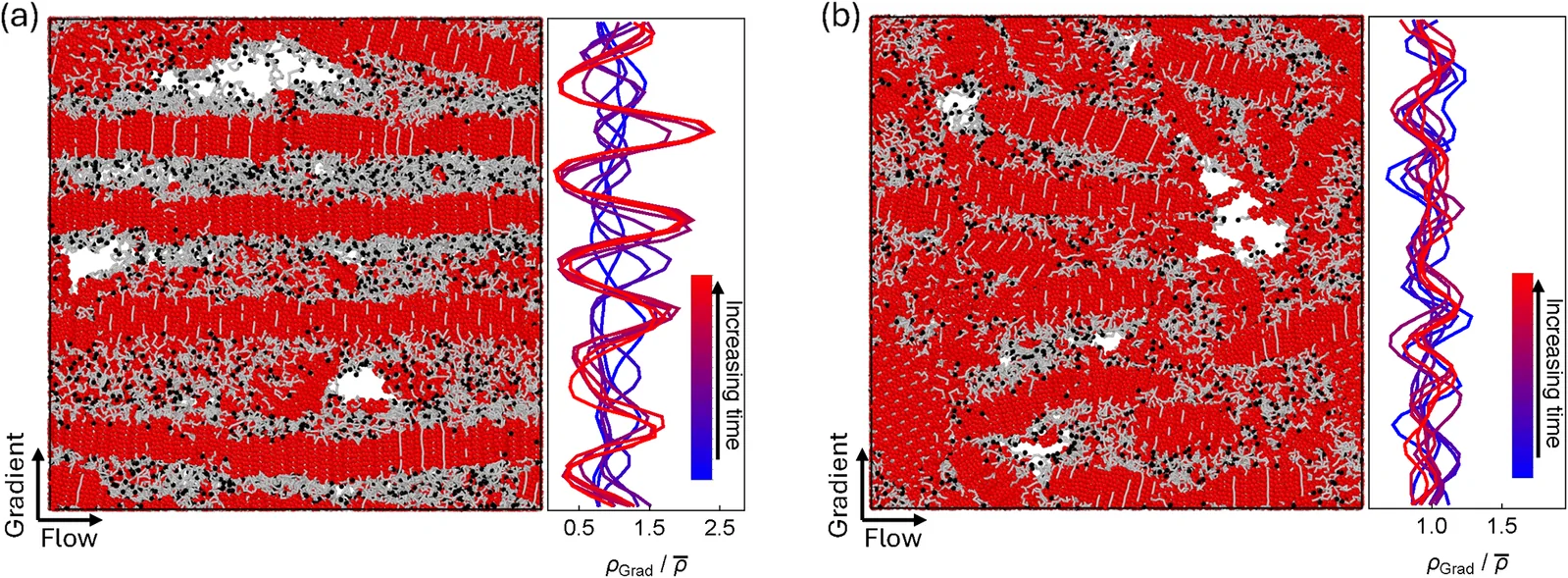
Shear-induced structural changes in samples with N40 and ρ ≈ 0.27. (a) A snapshot of the formation of shear bands at a low shear rate (*Wi* = 1) with the evolution of PR density over time. (b) A snapshot of the smaller crystalline domains at high shear-rate (*Wi* = 10) with the evolution of PR density.

To quantify the flow-induced structural rearrangement, we monitored the number of crystalline domains in PR samples during shear. Increasing shear rates from *Wi* = 1 to 10 resulted in more crystals due to rupturing the crystalline domains formed under quiescent conditions (Figures 10b and S7a). The initial increase in the number of crystalline domains under shear was followed by a subsequent decrease and plateau at or below the equilibrium value when *Wi* = 1 and 5. This is due to the merging of domains to form shear bands or periodic crystals. As two crystals need to align along the lamellae edge to merge, the shear-enhanced orientation at moderate flow rates (*Wi* = 1 and 5) facilitates the process. However, increasing shear to *Wi* = 10 resulted in more crystals during shear. The enhanced tumbling of the domains at the higher shear rate lowered the probability of two neighboring crystals achieving the ideal configuration for merging and prevented the formation of large domains.

**Figure 10:**
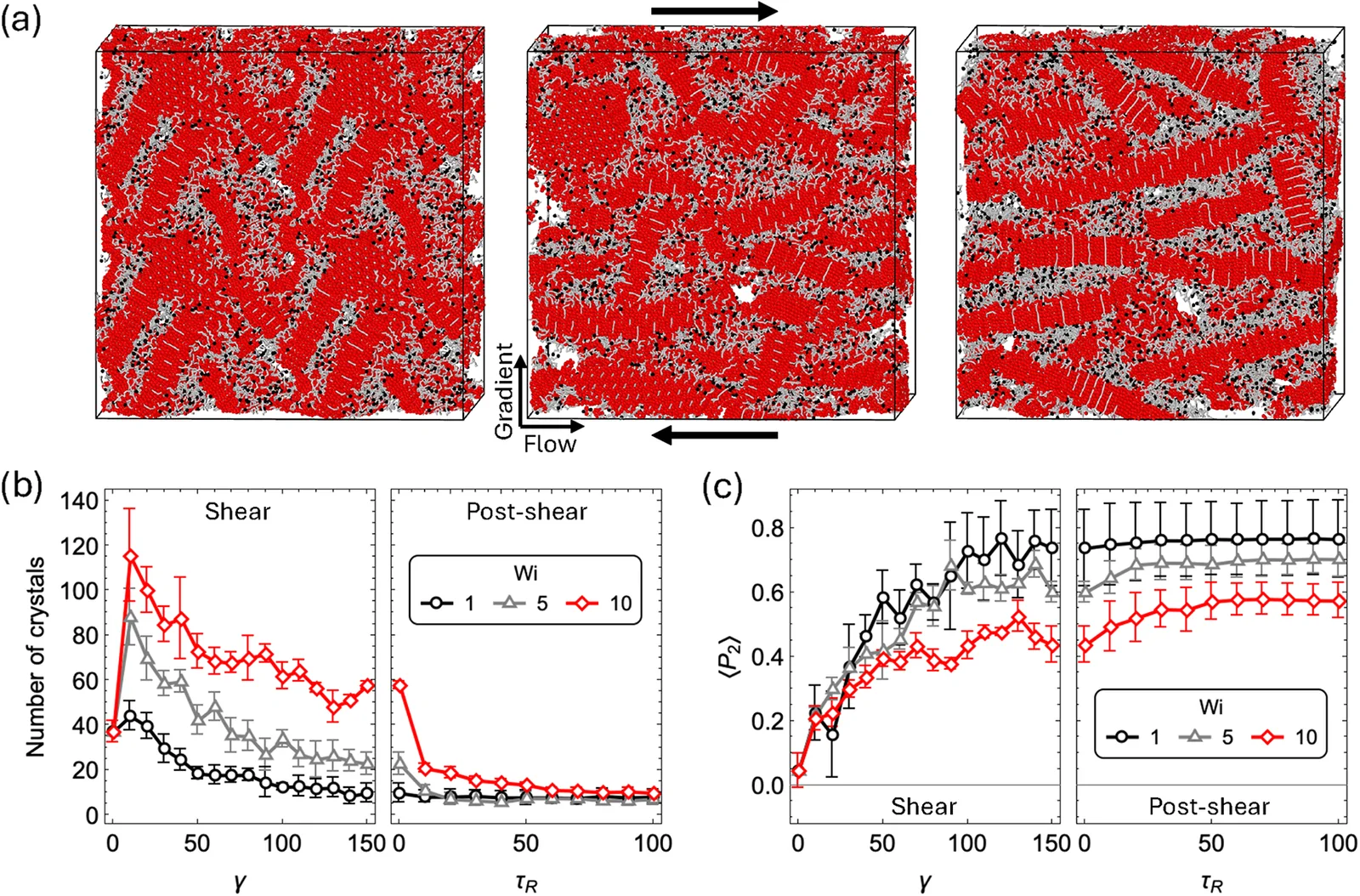
Shearing of N40 PR networks at ρ ≈ 0.27. (a) Snapshots of the system in quiescent, sheared (*Wi* = 10), and postshear states. Evolution of (b) number of crystals, and (c) orientational order of the crystals during shear and recovery. Orientational order (⟨*P*_2_⟩) is determined as the norm of the crystalline macrocycles with regards to the gradient direction.

We also quantify the flow-induced alignment of lamellae during shear using the orientational order (⟨*P*_2_⟩) of the crystalline macrocycles. Figure 10c (and Figure S7b) shows the initial crystalline domains orientated isotropically with ⟨*P*_2_⟩ ≈ 0.0. Exposure to shear results in a significant increase in anisotropy with ⟨*P*_2_⟩ plateauing around 0.7 and 0.6 for *Wi* = 1 and 5, respectively, and *Wi* = 10 reaching 0.4. The decrease in alignment with increasing shear rates is due to the enhanced tumbling of the domains and the reduced crystallite sizes.

The rupture of crystals formed under quiescent conditions also disrupts percolation in PR networks. To show this, we performed percolation analysis on the systems during the last 50γ of the shearing simulation, once the systems reached steady-state. We counted the snapshots containing system-spanning crystals or bridged clusters to obtain the percolated fraction (Figure 11). Percolation was significantly diminished in the gradient direction at all shear rates for the dilute system. The formation of shear bands at *Wi* = 1 resulted in the high fraction of percolation in the flow and vorticity directions; however, increasing the shear rate to *Wi* ≥ 5 suppressed the establishment of stable bands.

**Figure 11:**
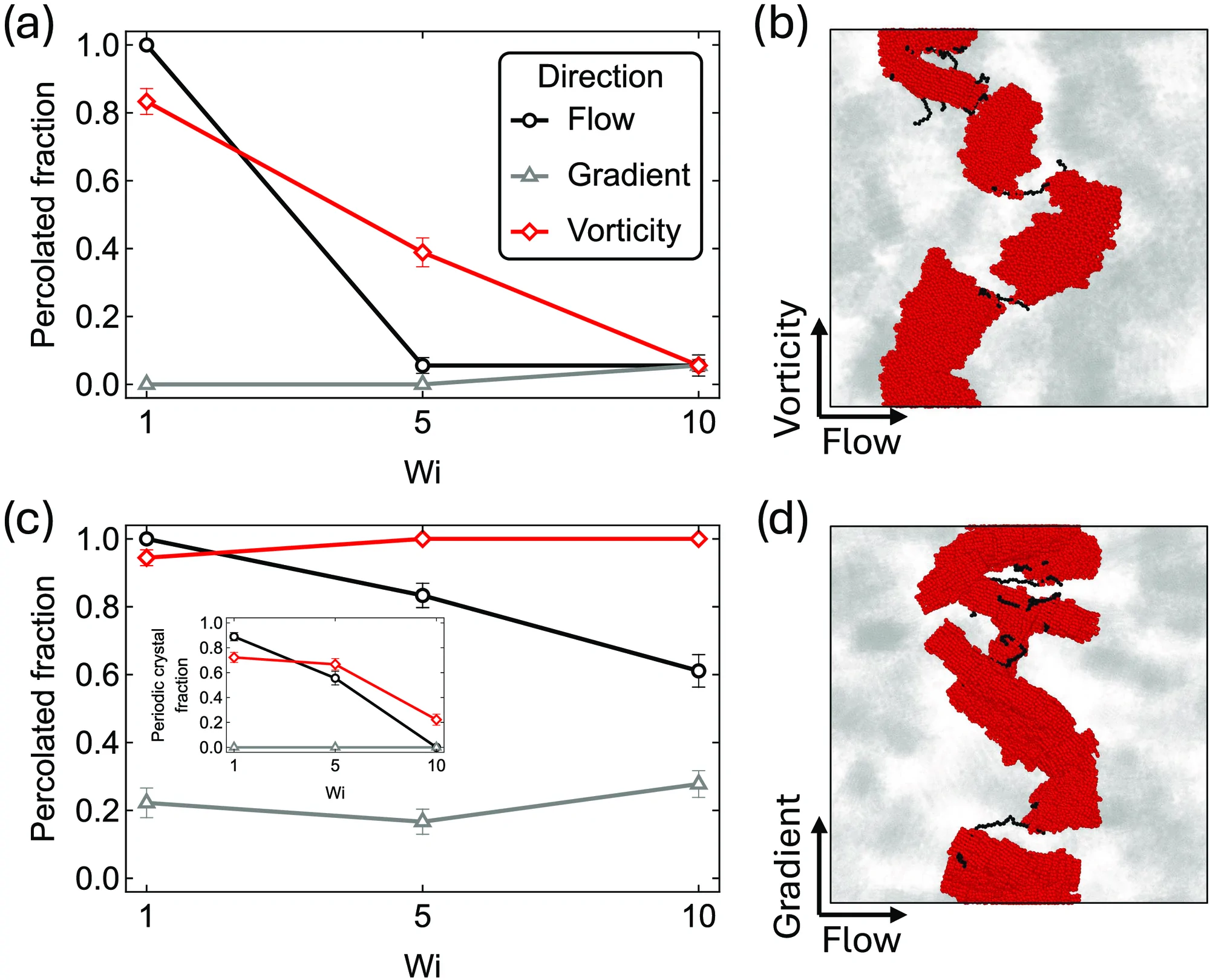
Fraction of percolated snapshots during shear for systems at ρ: (a) 0.18, and (c) 0.27. Inset in (c) shows the fraction of periodic crystals that account for percolation. Snapshots highlighting the crystalline domains and bridging polymers that enable percolation in the (b) vorticity, and (d) gradient direction at ρ ≈ 0.27 and *Wi* = 10.

At the higher concentration of ρ ≈ 0.27, the system exhibited an increased tendency to percolate in all dimensions during the shearing process at all shear rates. The formation of shear bands at *Wi* = 1 and 5 again result in persistent percolation in the vorticity and flow direction (Figure 11c inset). Interestingly, we see an increase in percolation in the vorticity direction when the formation of shear banding decreases. We also find that the system was partially percolated in the gradient direction at all shear rates, indicating a dynamic breaking and reestablishment of domain connectivity through the shearing process. Further investigation of the connectivity at these high shear rates showed that the percolation in the vorticity and gradient direction is formed through a small number of polymers that bridge crystalline domains (Figure 11b,d).

Nonetheless, the flow-induced structural change and the reduced percolation in the PR samples resulted in reduced shear viscosity (η), which we obtained using

10 $$\eta \left(\mathrm{\gamma ̇}\right)=\frac{\sigma_{\mathrm{xy}}\left(\mathrm{\gamma ̇}\right)}{\mathrm{\gamma ̇}}$$

where σ_*xy*_ is the steady-state shear stress, taken from the stress tensor. As expected, the more concentrated sample exhibits a higher shear viscosity and the reduced percolation in the PR samples results in shear thinning (Figure 12). The viscosity drops over the range of shear rates in agreement with experimental shear sweep profiles of PR hydrogels.^12,21^

**Figure 12:**
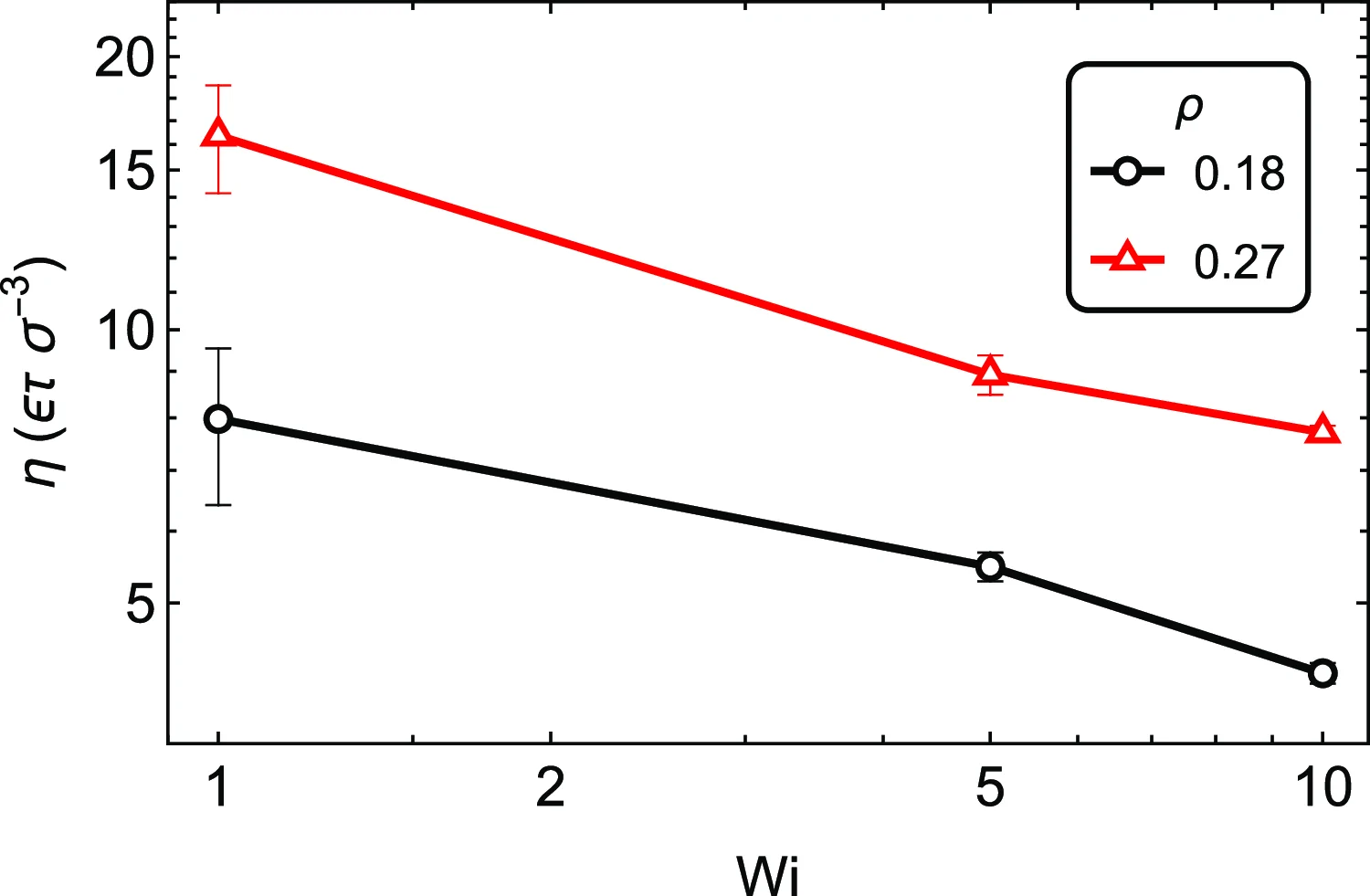
Viscosity vs shear strength in PR systems of two different concentrations.

### PR Network Recovery after Shear

The PR samples were re-equilibrated under quiescent conditions after exposure to shear flow to mimic the post-extrusion recovery. We observed rapid crystal merging across all samples prepared at high shear rates which resulted in a smaller number of larger crystals than those formed under quiescent conditions (Figures 10b and S7a). For samples processed under moderate shear flows, the minimal effect of the cessation of shear is attributed to the merging of crystals during processing. For samples processed under fast shear flow, the crystalline domains retained the pronounced flow-induced alignment, while the absence of collisions with tumbling neighboring domains following removal of the flow field facilitated crystallite fusion.

The flow-induced alignment also resulted in the formation of strongly anisotropic crystalline domains, with the normals of lamellae aligned in the gradient direction (Figures S19–S22). To quantify the effect of shear on the crystal orientation, we determined the ⟨*P*_2_⟩ of the crystalline macrocycles, relative to the gradient direction. The uniaxial alignment of lamellar normals in the gradient direction indicates that the long axes of the anisotropic PR crystallites point in the flow and vorticity direction. Figure 13a shows that the initial equilibrated domains oriented isotropically with ⟨*P*_2_⟩ ≈ 0.0. Exposure to shear at any rate causes a pronounced increase in anisotropic alignment of crystalline domains at both densities with ⟨*P*_2_⟩ ≈ 0.6.

**Figure 13:**
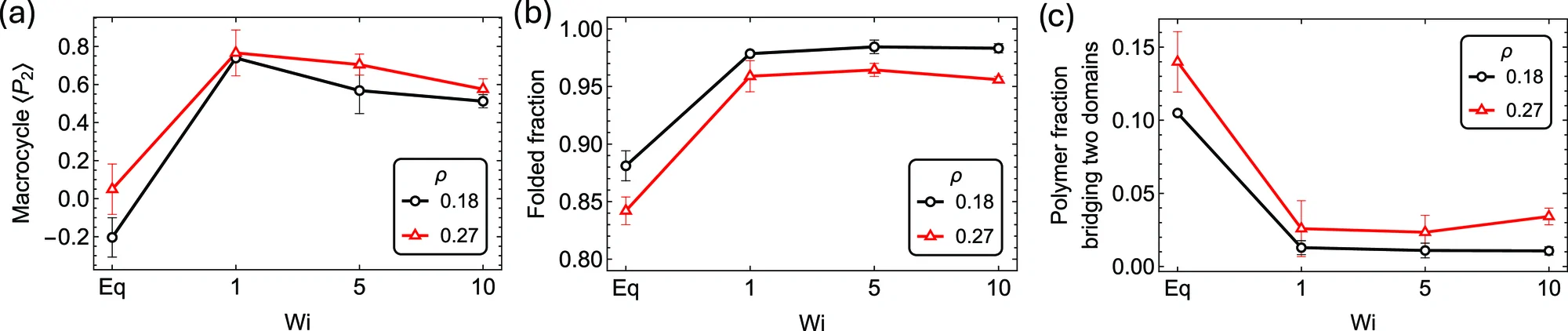
Effect of shear rates on crystalline domains after postshearing re-equilibration of N40 samples. (a) Orientation order of crystalline macrocycles in the gradient direction, (b) fraction of folded PR, and (c) fraction of polymers that bridge two domains.

We elucidated the fraction of stem and folded PR present in addition to the orientation of the crystalline domains (Figure 13b). Exposure to shear dramatically increased the presence of folded PR as a result of crystal merging, either during the shearing process at *Wi* = 1, or after recrystallization when *Wi* = 10. This was further supported by the loss in population of polymers that bridge two crystalline domains (Figure 13c). Concentrated systems retained a slightly lower composition of folded PR and more polymers bridging two domains. Nonetheless, in all the samples, less than 5% of the total polymers act as bridges between crystalline nodes.

The formation of domain connectivity through bridging polymers is more likely to occur at the edge of a PR crystal because it requires the PR to unfold from the crystallite and attach to the edge of a neighboring crystal. Due to the formation of larger crystals during or after shear, there is a decrease in crystal edges that can facilitate the establishment of bridging polymers. Therefore, we observe a reduction in domain connectivity. As the long axes of the lamellae are highly aligned in the flow and vorticity direction, the effect of diminished domain bridging is expected to have a pronounced effect on the connectivity and stress transmission in the gradient direction.

The flow-induced morphology changes indeed impact the mechanical properties of the PR networks. To show this, we quantified the material properties of the PR hydrogels using the stress relaxation modulus (*G*(*t*))

11 $$G\left(t\right)=\left\langle \sigma_{\mathrm{ij}}\left(t\right)\sigma_{\mathrm{ij}}\left(0\right)\right\rangle$$

where *ij* ⊂ {*x*, *y*, *z*}. The stress correlations were measured from additional simulations of 50τ_*R*_ for the equilibrated and recrystallized systems using the multiple-tau correlator method.^91^ For samples that crystallized under the quiescent conditions from isotropic solutions, we averaged *G*(*t*) over all directions and configurations to reflect the isotropic orientation and connectivity of the PR crystallites in the networks. For the recrystallized systems, we present *G*(*t*) in the *xz* plane to demonstrate the effect of the formation of shear bands and large lamellae, and combine *G*(*t*) in the *xy* and *yz* planes to illustrate the impact of diminished connectivity in the gradient direction. Additional *G*(*t*) plots at each shear rate and all dimensions are available in the SI (Figures S9–S10).

Significant morphological changes in the underlying structure manifest in highly anisotropic stress relaxation profiles after re-equilibration, yet most samples exhibit plateaued modulus, which indicates the formation of gels. For the concentrated system, the shear bands formed at *Wi* = 1 crystallized into 2D lamellae and resulted in a *G*_*xz*_(*t*) plateau greater than that of the presheared sample (Figure 14a). Increasing the shear rate to *Wi* = 5 had a mild effect on band formation during shear. However, the highly aligned domains rapidly established crystals during the postshear processing to allow 2D sheet formation in the flow and vorticity (*xz*) directions, and in turn yield a plateau modulus approximately equivalent to that of the presheared sample. Shear banding was not observed when *Wi* = 10 and the small crystallites did not permit well-established percolation in *xz* after shear. As a consequence, *G*_*xz*_(*t*) for samples processed at *Wi* = 10 lacks a well-defined rubbery plateau in the stress relaxation. The observed trend in *G*_*xz*_(*t*) is consistent with the fraction of 2D lamellae formed in the re-equilibrated samples (Figure S8).

**Figure 14:**
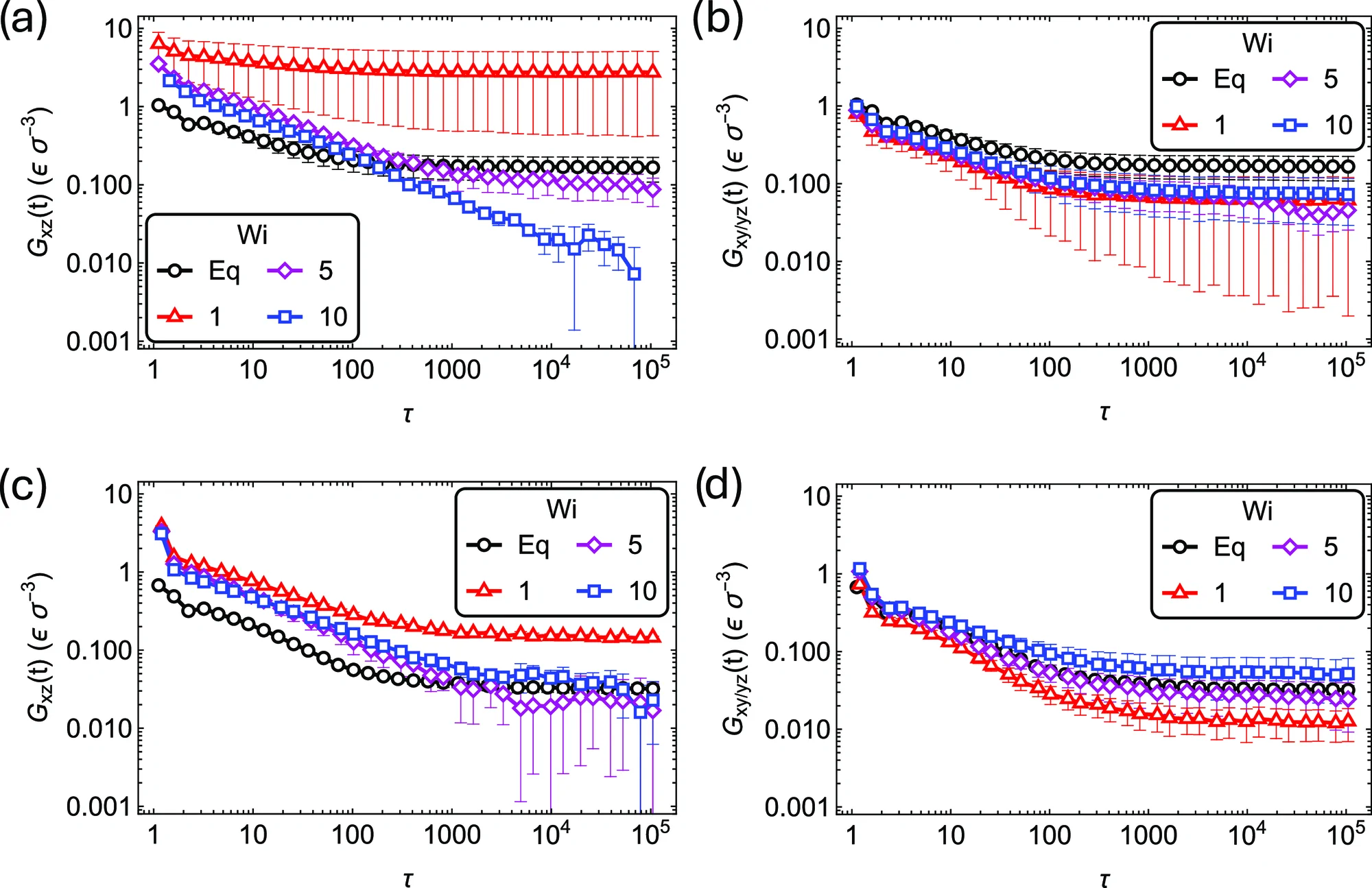
Stress relaxation modulus (*G*(*t*)) from the CG model at ρ ≈ 0.27 in the (a) *xz* plane, and (b) *xy*/*yz* planes. At ρ ≈ 0.18 in the (c) *xz* plane, and (d) *xy*/*yz* planes. Error bars represent the standard error obtained from simulations with distinct postshear initial configurations.

The formation of highly aligned lamellae at all shear rates resulted in the reduction of connectivity through the gradient direction after recrystallization. This loss of bridge formation led to a *G*_*xy*/*yz*_(*t*) plateau below that of the presheared samples at ρ ≈ 0.27 (Figure 14b). This is consistent with the formation of a softer gel after exposure to shear. Connectivity in the gradient direction is hindered by the substantial reduction in the fraction of bridging polymers and crystal edges. Because short chains are only capable of bridging two domains (Figure 5), network formation requires a sufficiently high concentration of bridging chains. Upon shear-induced rupture of crystallite-bridging polymers, the detached chains become incorporated into the domain associated with the tethered end, resulting in an increased fraction of folded PR chains. Therefore, insufficient bridging chains remain available to re-establish the extensive percolation observed during isotropic crystallization after removal of the applied shear stress, leading to a softer gel.

The isotropically crystallized sample in the dilute system exhibits a lower plateau than the concentrated sample, as expected. The trend in *G*(*t*)_*xz*_ profiles of the dilute system after recrystallization matched those at a higher concentration, with the formation of shear banding at *Wi* = 1 resulting in a higher plateau than the presheared sample (Figure 14c). Whereas high shear rates inhibited band formation and produced gels equivalent to or slightly softer than those formed in isotropic crystallization. There was a less pronounced effect on *G*(*t*)_*xy*/*yz*_ after recrystallization than in the concentrated system, with stiffness roughly equivalent or slightly softer than the presheared sample.

Nevertheless, our simulations demonstrate that shear flows can affect the crystalline morphology and result in anisotropic mechanical properties in PR gels. Our finding here is consistent with experiments. For example, experimental step-strain sweeps of PR hydrogels, even those successfully used as 3D printing inks, show a decrease in modulus as a result of poor self-healing capabilities.^3,10,12^ As a consequence, current methods for the utilization of PR hydrogel inks in extrusion-based 3D printing undergo a postprinting covalent cross-linking procedure to form the final gel.^10,12^ Our results reveal that the decrease in modulus and requirement for postdeposition treatment is likely due to the loss of domain connectivity after exposure to shear.

Our analysis of the connectivity between crystallites indicates that increasing the population of dangling or free PR by breaking large crystals during shear could facilitate the reformation of a more robust gel after printing. This could be accomplished using a cosolvent, such as hydrogen-bond-breaking dimethyl sulfoxide (DMSO) to disrupt the αCD crystal,^22^ or increasing the printing temperature. Exploring the optimization strategies for better 3D-printing PR hydrogels, however, is beyond the scope of this paper and is warranted for future work.

## Conclusions

We have employed coarse-grained simulations to investigate the crystallization in hydrogel-forming PR samples, which are promising materials for 3D-printing applications. Our CG simulations recovered the experimental crystalline morphology of PR solutions and gels at varying concentrations and chain lengths by observing the formation of 2D stem lamellae, folded lamellae, and bridged clusters. Together with nonequilibrium simulations of PR gels under shear flows, our model elucidated the effect of concentration and shear rate on the crystalline domains formed before, disrupted during, and reformed after shear stress imposed by extrusion-based 3D printing.

We show that exposure to a moderate shear flow can result in broken crystalline domains, which then merge to form shear bands and highly aligned lamellae that are stacked in the gradient direction. Increasing the shear rate mitigated the band formation, yet highly anisotropic crystals still formed after recrystallization due to increased crystalline alignment. At all shear rates, the lack of available dangling PR chains and reduction of lamellae edges reduced the reestablishment of connectivity in the gradient direction and resulted in the formation of softer gels than the presheared samples. These results explain the decrease in modulus observed in experimental step-strain experiments as a loss in domain connectivity. We speculate that increasing free PR during extrusion would enable the domains to re-establish domain connectivity after printing and result in a stiffer gel. Overall, we expect our simple CG model to help better design and optimize PR-based hydrogels for 3D printing applications.

## Supplementary Material



## Notes

Details concerning the parametrization of the coarse-grained polyrotaxanes, relaxation times, equilibration, multiparticle collision dynamics comparison, additional stress correlation plots, density profiles, and snapshots of each equilibrated system (PDF)
