# Supplementary material for: Coarse-Grained Simulations of Polyrotaxane Hydrogels under Quiescent and Shear Conditions
Source: Macromolecules. 2026 Jul 8;59(16):9124–36. doi: 10.1021/acs.macromol.6c00624 (PMC13520876; doi:10.1021/acs.macromol.6c00624)
Supplement: Supplementary file 1 [file ma6c00624_si_001.pdf]

Supplementary Information: Coarse-grained simulations of polyrotaxane hydrogels under quiescent and shear conditions

Cameron D. Smith and Wenlin Zhang*

Department of Chemistry, Dartmouth College, Hanover, New Hampshire 03755, USA

E-mail: wenlin.zhang@dartmouth.edu

Contents

1 Matching experimental concentrations 3

2 Macrocycle attraction 4

3 Relaxation times 5

4 Orientational order (S) and crystallinity 6

5 Equilibration in quiescent conditions 7

6 Hydrodynamic interactions in shear simulations 8

7 Shearing and recovery of the dilute system 10

8 Formation of 2D crystals after shear 10

9 Stress correlation 11

10 Equilibrated system snapshots 12

11 Sheared system snapshots and density profiles 16

12 Re-equilibrated system snapshots 20

1 Matching experimental concentrations

The experimental concentrations were approximated by determining the concentration of Kuhn segments, or beads, of polyethylene oxide (PEO) in solution, where one Kuhn segment is about three monomers.[1] Number densities of 0.13 σ^{-3} and 0.27 σ^{-3} were determined for the crystal morphology[2] and 3D printing studies,[3] respectively. Each α-cyclodextrin (αCD) occupies two ethylene glycol monomers in a polyrotaxane and the coverage ranged from approximately 30 - 60 % in the experimental studies.[3] An αCD molecule occupies approximately 1 nm^3 of volume[4] which matches the Kuhn segment of PEO, however, the macrocycles in this coarse grained model are comprised of six beads. Therefore, we swell the box dimensions to retain the approximate number densities of PEO Kuhn segments in solution to account for the disparity in size.

2 Macrocycle attraction

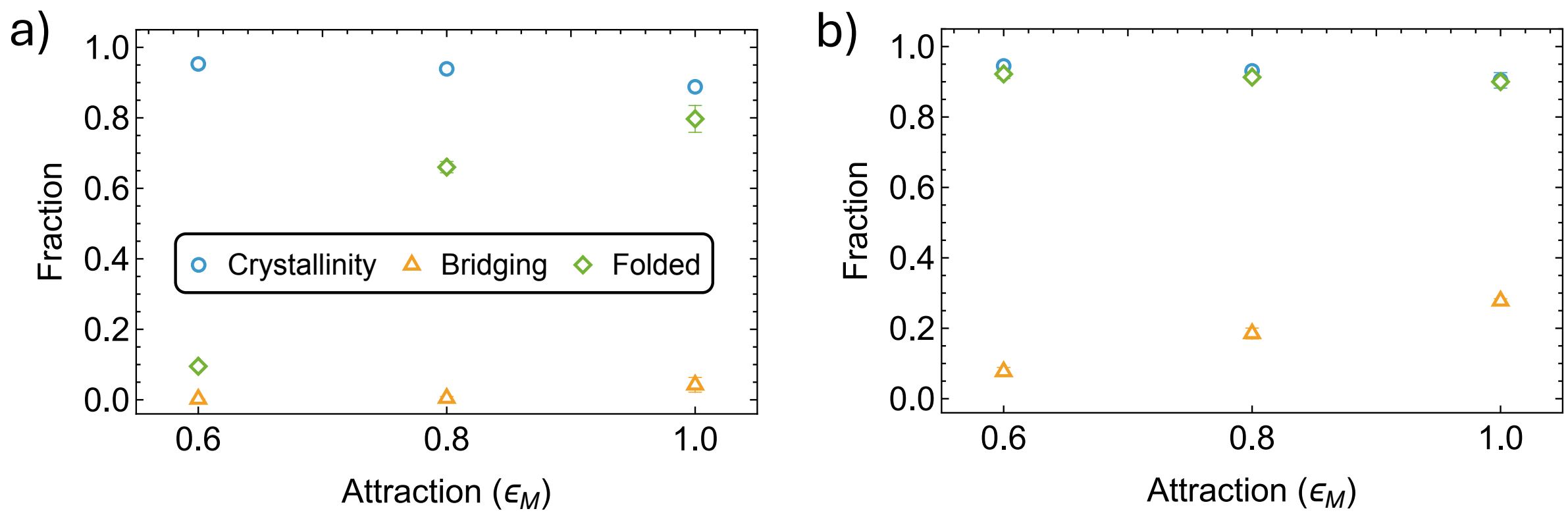

Figure S1: Effect of macrocycle attraction (ϵ_M) on crystallinity, bridging fraction, and folded content at $\rho \approx 0.13$ for chain lengths: a) N20 and, b) N40.

The attraction between macrocycles (ϵ_M) was varied from $0.6 - 1.0\ \epsilon$ to determine that which most accurately recovered the experimental characterization at $\rho \approx 0.13$.[2] All attraction strengths resulted in highly crystalline systems, with every system reaching crystallinities greater than 0.9. There was a significant effect on the crystalline morphology spanning the attractive values. Short chains (N20) with high attraction formed highly folded crystalline domains (Fig. S1a), whereas reducing the attraction to 0.6ϵ resulted in a stem fraction of over 0.9, and a high stem fraction for the N20 chain is in agreeance with the experimental characterization. Domain connectivity was also sensitive to the attraction strength, with $\epsilon_M = 0.6\epsilon$ having a bridging fraction of less than 0.1 at N40 (Fig. S1b), when we expect to form isolated nanosheets with a high folded content. We therefore use an attraction of $\epsilon_M = 0.6\epsilon$ throughout the study as this recovers the experimentally determined morphology.

3 Relaxation times

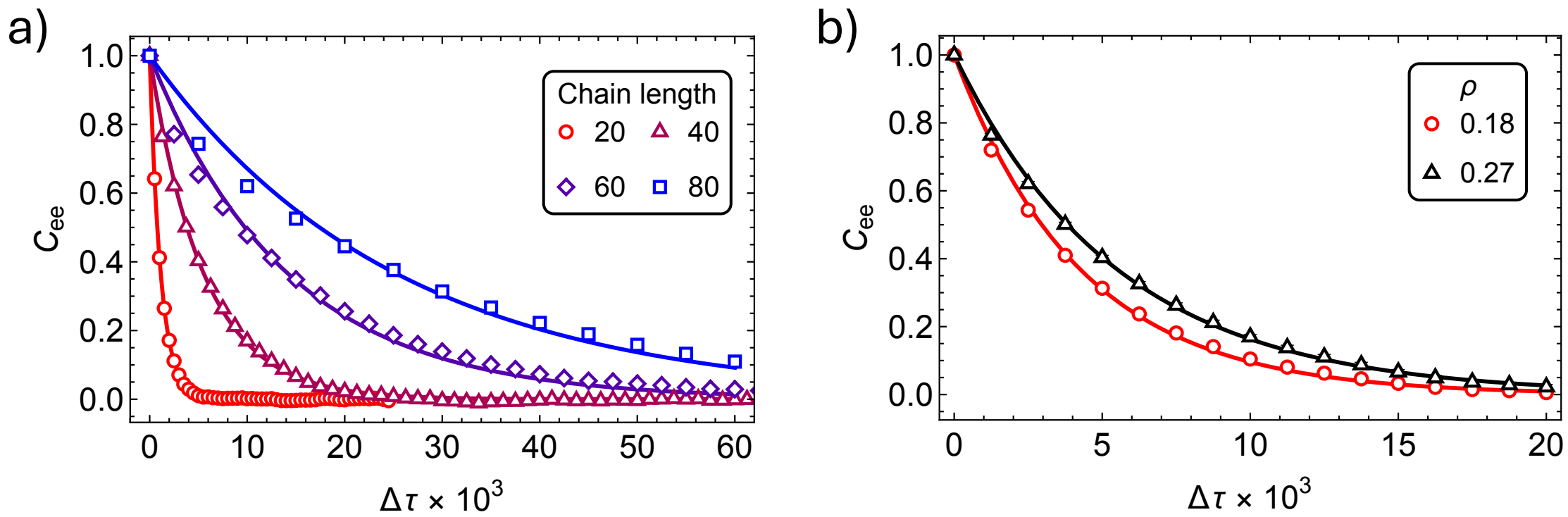

Figure S2: Autocorrelation functions of the end-to-end vector. a) Increasing chain length for the most concentrated systems ($\rho \approx 0.27$) in quiescent conditions. b) Concentration effect on relaxation at chain length N40. Symbols show the simulation data with the standard error of the mean being smaller than the symbols. The solid lines show the fitting.

The relaxation time (τ_R) of each chain length was determined using the autocorrelation function of the end-to-end vectors:

$$C_{ee} = \frac{\langle R_{ee}(\Delta t) - \bar{R} \rangle \langle R_{ee}(0) - \bar{R} \rangle}{\langle (R_{ee}(0) - \bar{R})^2 \rangle} \tag{1}$$

where R_{ee} and $\bar{R}$ are the end-to-end vector and average end-to-end vector, respectively, and $\langle \cdots \rangle$ indicates the ensemble average. The data were fit using $C_{ee} = Exp[-t/\tau_R]$ to approximate τ_R (Fig. S2a). We obtain τ_R of 1100, 5500, 14000, and 25000 τ for chain lengths of 20, 40, 60, and 80, respectively.

To determine the shear rate required to match Wiessenberg numbers (Wi), where $Wi = \dot{\gamma}\tau_R$, we determined the τ_R at $\rho \approx 0.18$ and 0.27 for chain length of 40 (Fig. S2b). We establish τ_R of 4000 and 5500 for $\rho \approx 0.18$ and 0.27, respectively.

4 Orientational order (S) and crystallinity

Crystallinity was determined using the local orientational order (S) of macrocycle aggregates. We determined S for the macrocycles in the perfect crystal and the WCA solutions at $\rho \approx 0.27$ to identify a suitable cutoff value (Fig. S3). A value of $S \geq 0.9$ was chosen to identify crystalline macrocycles.

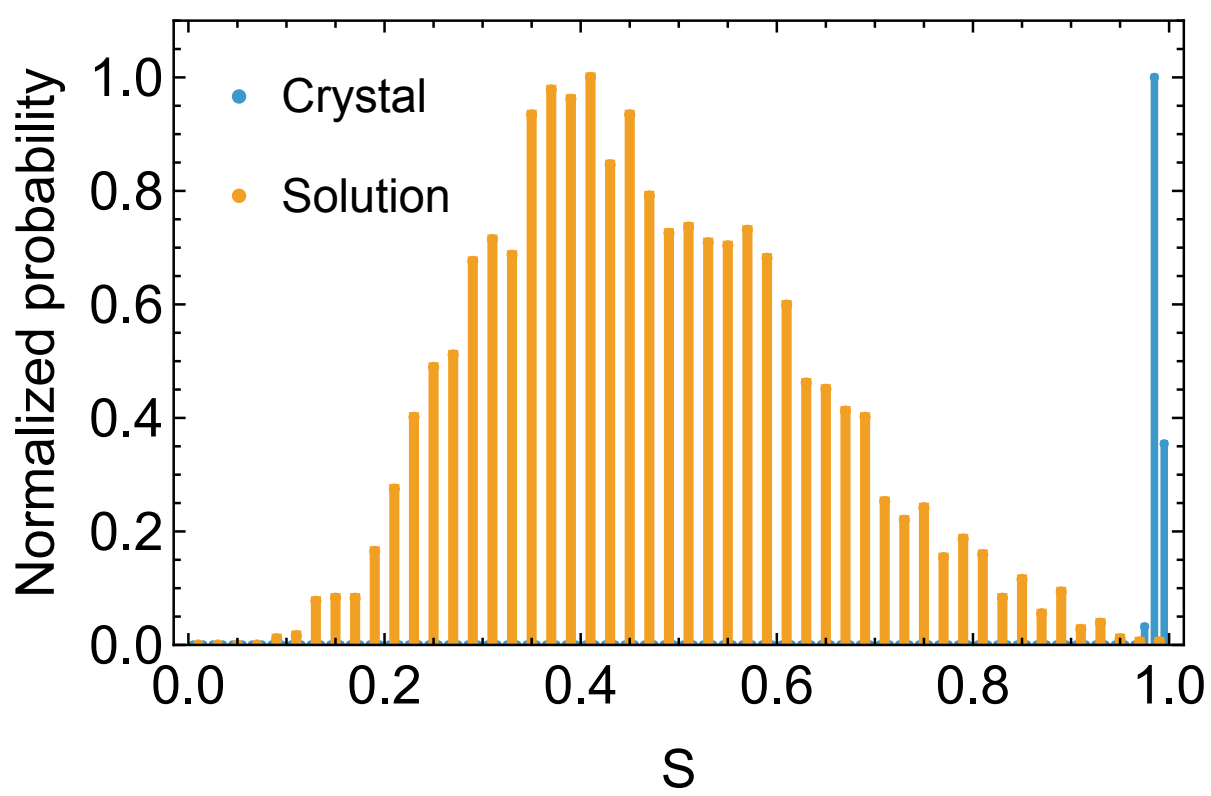

Figure S3: Local orientational order (S) for the crystal slab and WCA solutions.

5 Equilibration in quiescent conditions

Monitoring the evolution of crystallinity (Fig. S4) and number of crystalline domains (Fig. S5) during crystallization in quiescent conditions.

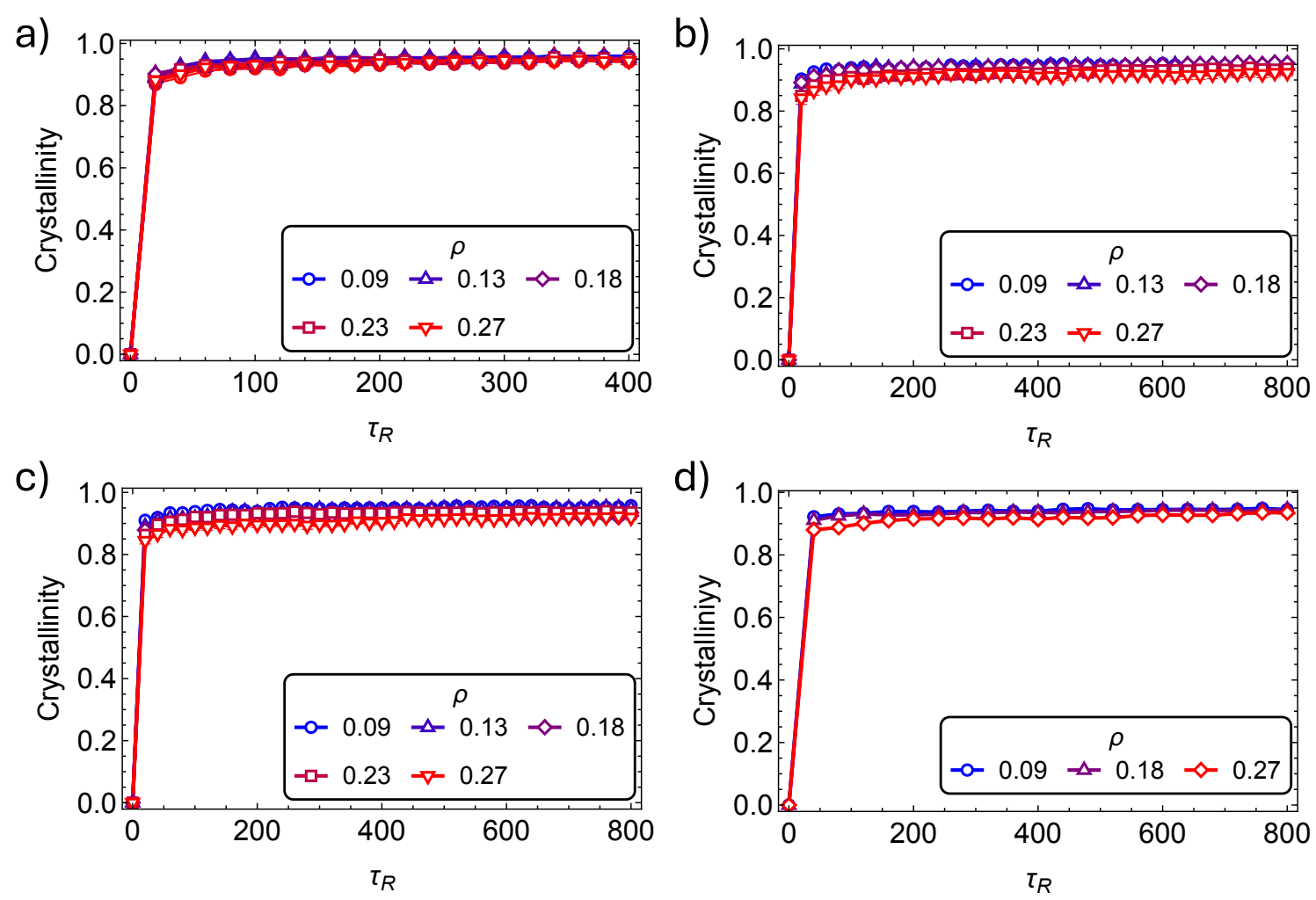

Figure S4: Crystallinity in the system during equilibration at chain length: a) 20, b) 40, c) 60, and d) 80.

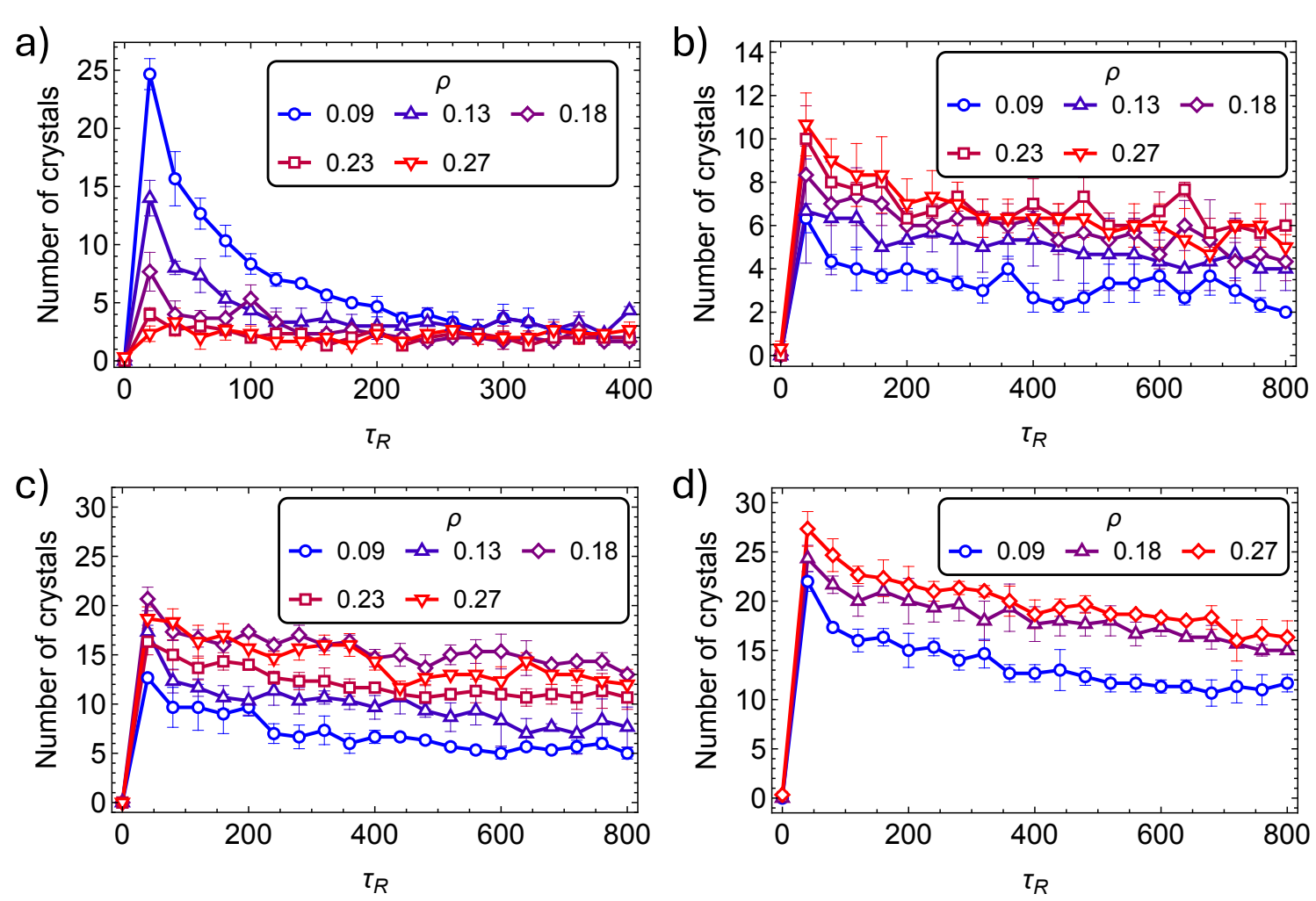

Figure S5: Number of crystalline domains in the system during equilibration at chain length: a) 20, b) 40, c) 60, and d) 80.

6 Hydrodynamic interactions in shear simulations

To determine if the absence of hydrodynamic interactions was causing the formation of the "log-rolling" crystalline domains in the shear simulations, we utilized the hybrid multi-particle collision dynamics (MPCD)[5–7] with the Anderson thermostat collision rule using HOOMD-blue (version 6.1.1).[8] MPCD introduces the effect of HI on the solute through the incorporation of explicit point particles of mass m_s which undergo alternating streaming and collision steps. The intermolecular interactions between MPCD solvent particles are ignored, and the solvent undergoes simple ballistic streaming to update the position ($\mathbf{x}$) of particle i through:

$$\mathbf{x}_i(t + \Delta t_{MPCD}) = \mathbf{x}_i(t) + \dot{\mathbf{x}}_i(t)\Delta t_{MPCD} \tag{2}$$

where Δt_{MPCD} is the time between collisions.

At each collision step (every Δt_{MPCD}) the solute and solvent particles are sorted into voxels of side length a and center of mass velocity ($\mathbf{v}_{com}$) of each voxel is determined. The velocity of particle i is then updated with:

$$\dot{\mathbf{x}}_i^{new} = \mathbf{v}_{com}(t) + \dot{\mathbf{x}}_i^{rand} - \mathbf{v}_{com}^{rand} \tag{3}$$

where $\dot{\mathbf{x}}_i^{rand}$ is a random velocity sampled from a Maxwell-Boltzmann distribution, and $\mathbf{v}_{com}^{rand}$ is the center of mass velocity of all random velocities assigned in the voxel.[9,10] The Verlet integrator was used to update the positions and velocities of the solute (PR) between the MPCD collision steps.

We set the solvent mass $m_s = 0.2\ m$, solvent density to $\rho_s = 5\ m_s\ a^{-3}$, and voxel side length $a = \sigma$ to match the average solvent mass per voxel.[6] We define the characteristic time in our MPCD simulations (denoted as τ_s to prevent confusion with the Langevin simulations) as $\tau_s = (m_s\sigma^2/\epsilon)^{1/2}$. We set the MD timestep to 0.01 τ_s and performed the collision step every $\Delta t_{MPCD} = 0.1\ \tau_s$. These parameters resulted in a solvent viscosity of $\eta \approx 3.7\ \tau_s k_B T$

σ^{-3} and Schmidt number (Sc), where $Sc = \nu/D$, of $Sc \approx 10$.[11,12]

Select configurations of equilibrated PR systems were solvated with MPCD particles and allowed to equilibrate for 1000 τ_s. Shear was then applied to the MPCD solvent using the Müller-Plathe method,[13,14] resulting in x, y, and z being the flow, gradient, and vorticity directions, respectively. The voxels were randomly shifted to ensure Galilean invariance.[15]

A shear rate was chosen to match Weissenberg number (Wi), where $Wi = \dot{\gamma}\tau_{MPCD}$, between the Langevin and MPCD methods. Each sample was sheared at $Wi \approx 10$ and τ_{MPCD} is determined in the same manner as τ_R except the PR are in MPCD under quiescent conditions.

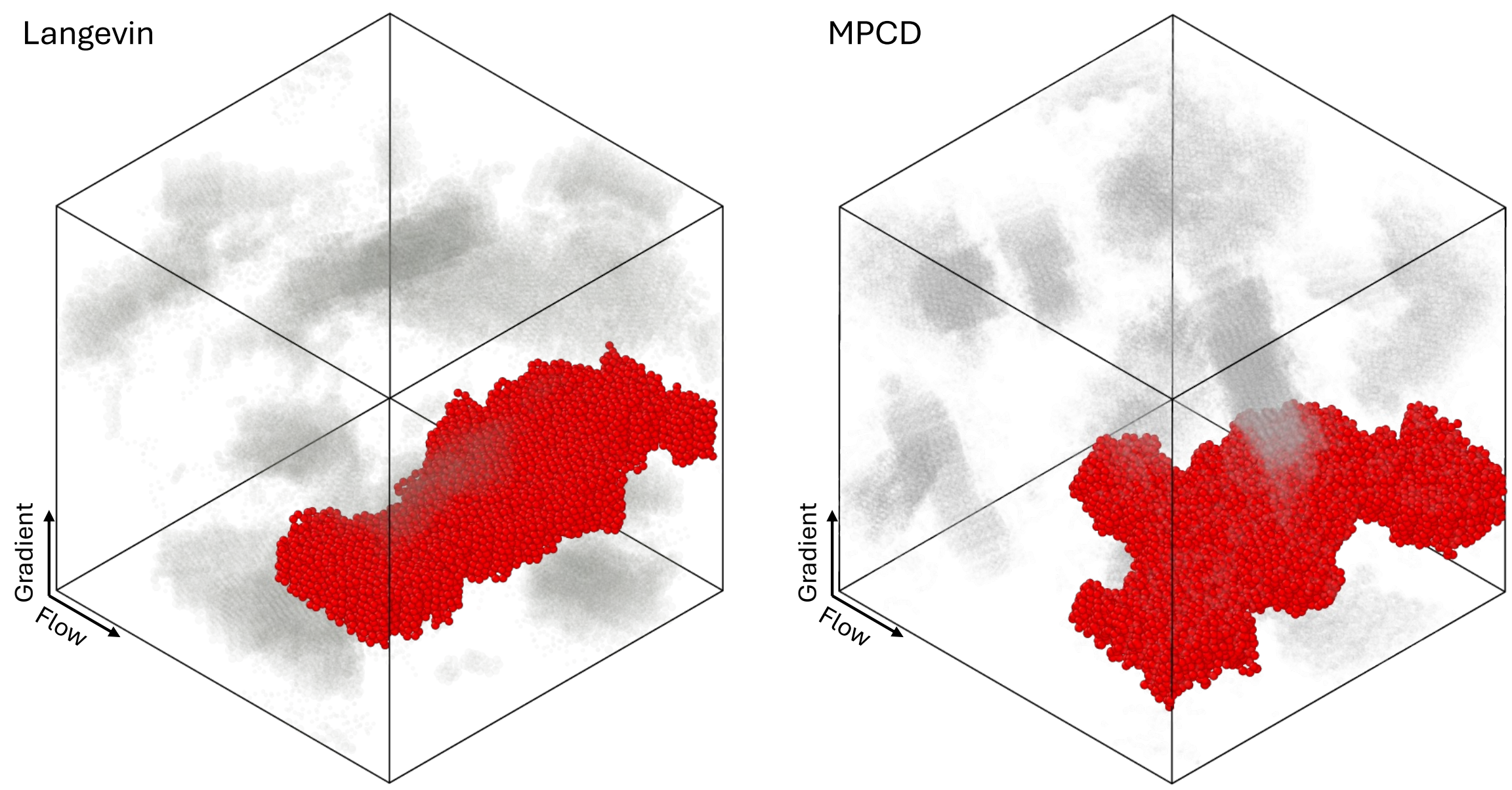

Figure S6: Formation of periodic crystals in the vorticity direction during shear using implicit Langevin dynamics and MPCD.

We observed the formation of a periodic crystal in the vorticity direction when shearing the system using MPCD and the Müller-Plathe method (Fig. S6). This indicated that the absence of HI in the Langevin shear simulations was not the cause of the structure and allowed us to determine that the formation of these unphysical crystals was due to the finite size of the box.

7 Shearing and recovery of the dilute system

Monitoring the number of crystalline domains and orientation during shear and recovery of the $\rho \approx 0.18$ systems (Fig. S7). Samples at $Wi = 5$ and 10 were sheared to a total strain of $\gamma = 200$ due to the slower onset of a steady state.

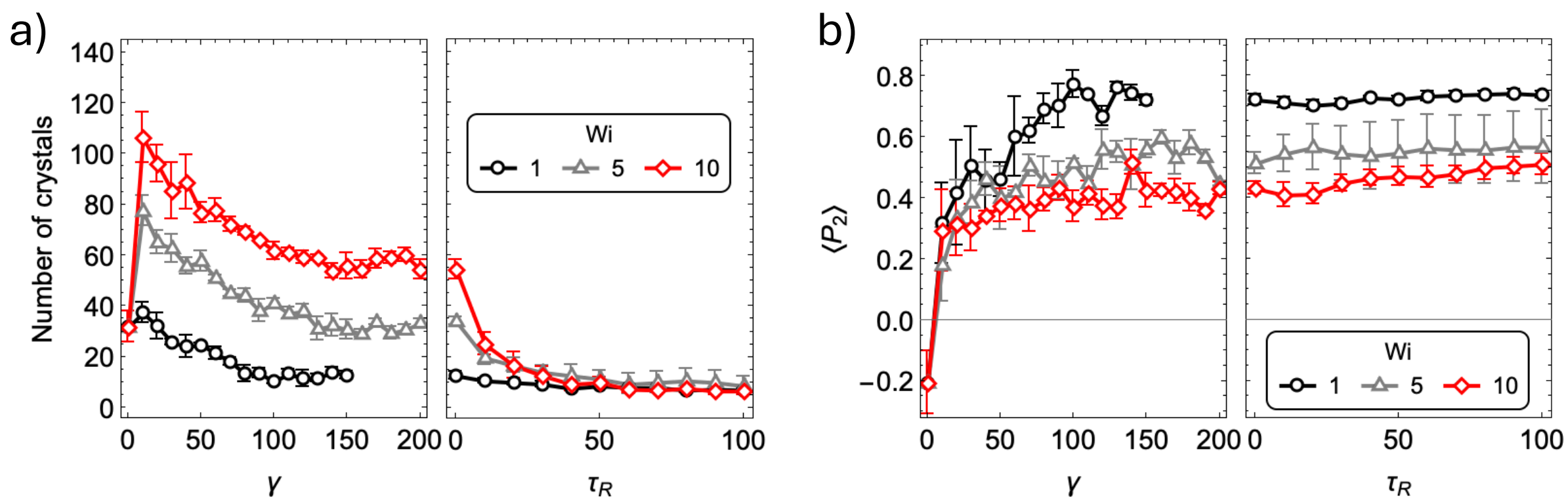

Figure S7: Shear and recovery of N40 PR at $\rho \approx 0.18$. Evolution of a) number of crystals, and b) orientational order, relative to the gradient direction, during shear and recovery.

8 Formation of 2D crystals after shear

Identifying the configurations that contain at least one 2-dimensional crystalline sheet in the sample after exposure to shear (Fig. S8).

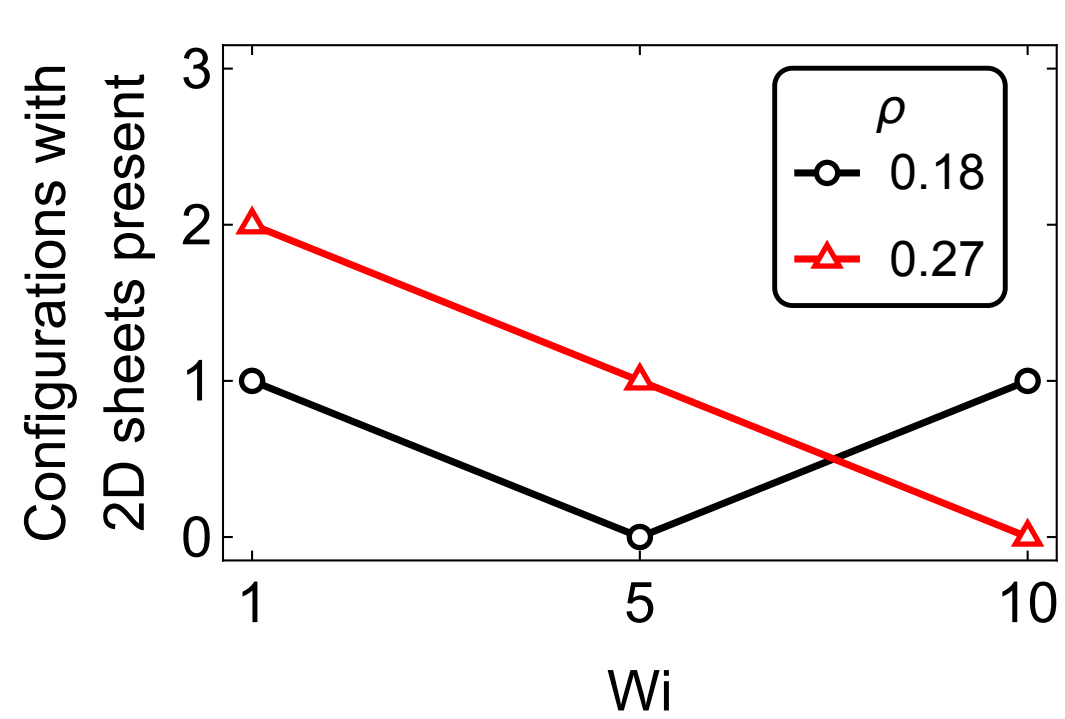

Figure S8: Number of configurations that contain a 2-dimensional crystal after recrystallization.

9 Stress correlation

Additional stress relaxation moduli $G(t)$ plots for each sample (Fig. S9-S10).

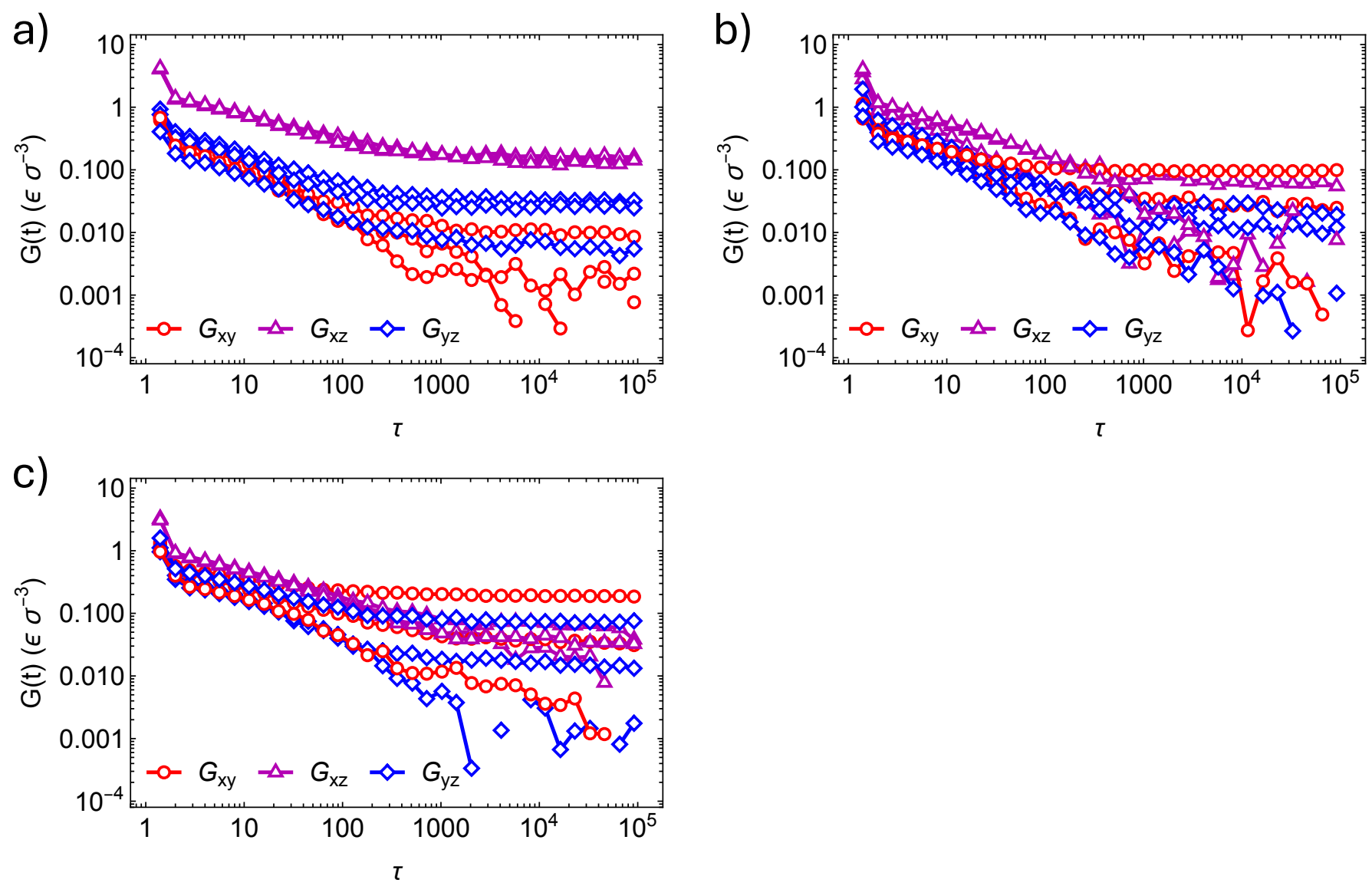

Figure S9: Stress relaxation modulus ($G(t)$) from the CG model after shear and re-equilibration for $\rho \approx 0.18$. Results for Wi: a) 1, b) 5, and c) 10.

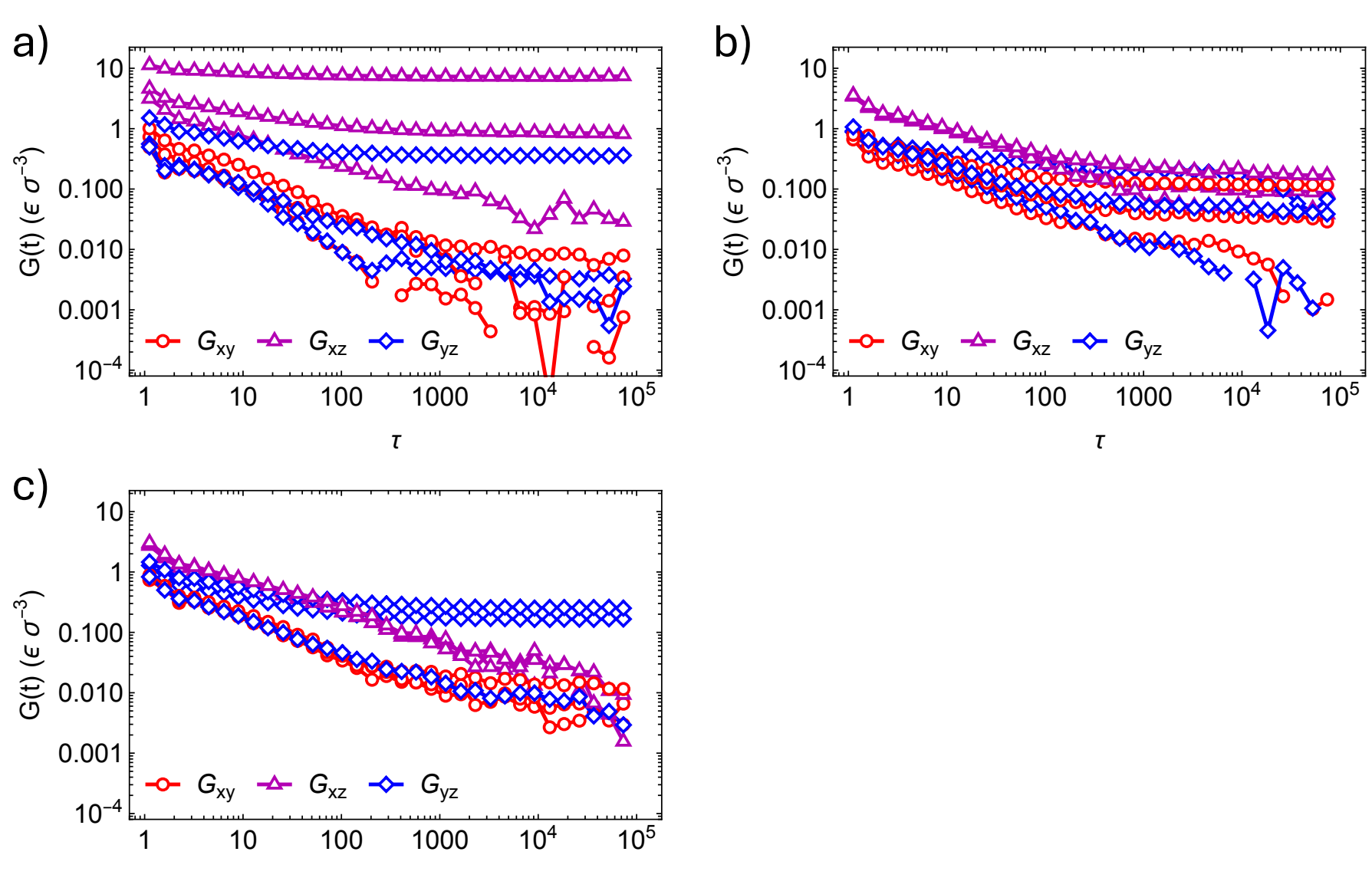

Figure S10: Stress relaxation modulus ($G(t)$) from the CG model after shear and re-equilibration for $\rho \approx 0.27$. Results for Wi: a) 1, b) 5, and c) 10.

10 Equilibrated system snapshots

Final snapshots of each system at the end of equilibration (Fig. S11-S14).

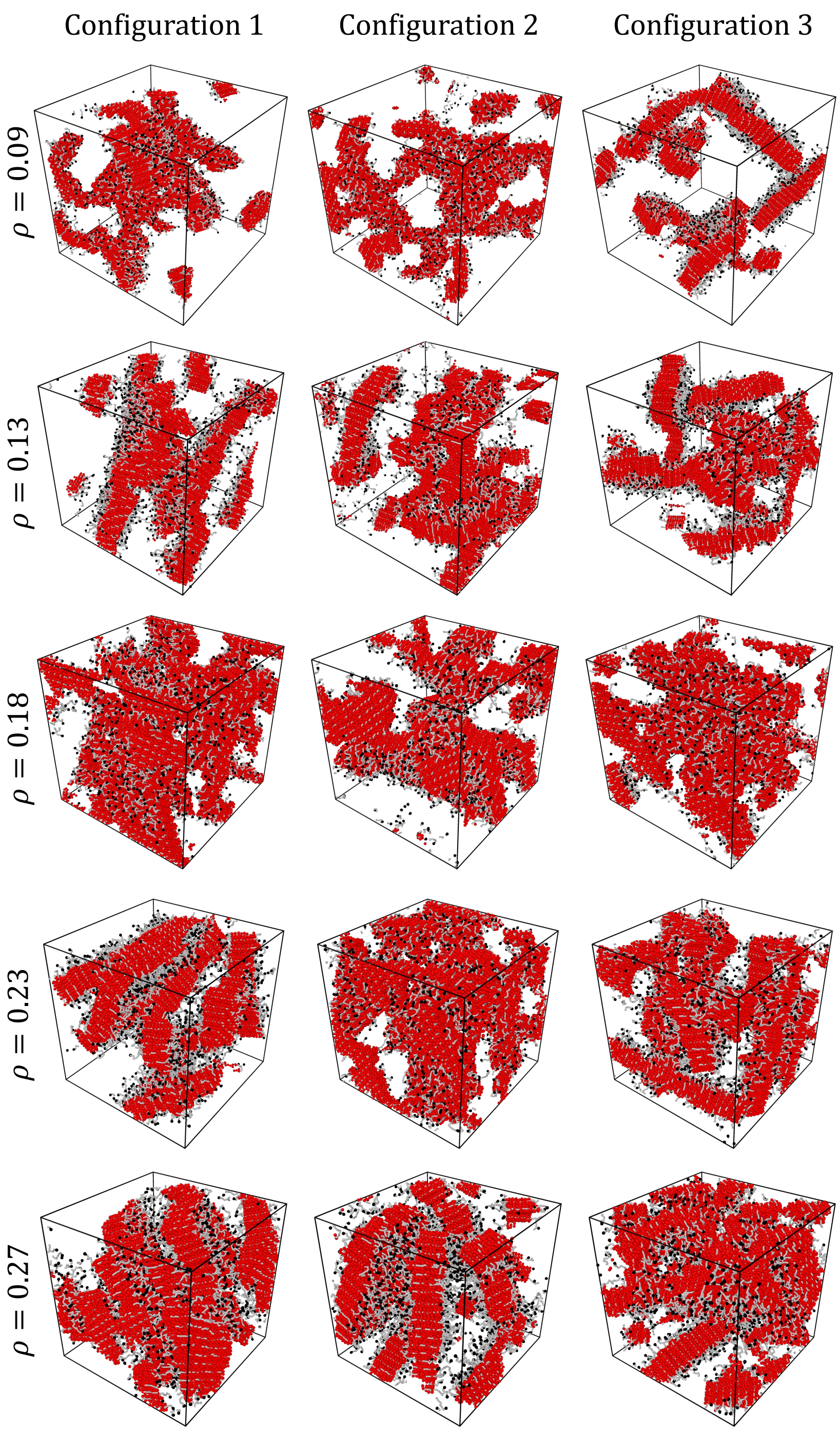

Figure S11: Equilibrated N20 systems crystallized under quiescent conditions

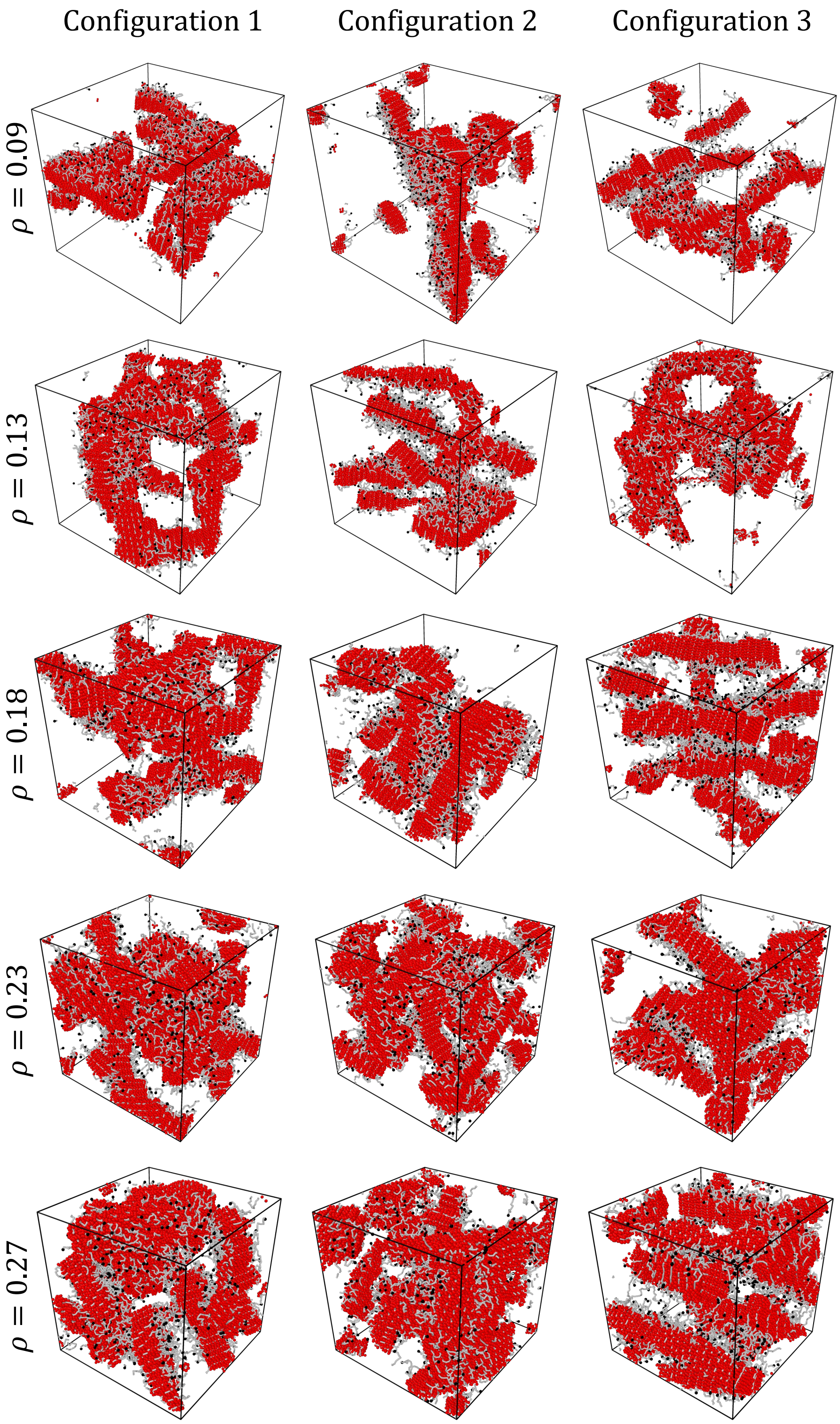

Figure S12: Equilibrated N40 systems crystallized under quiescent conditions

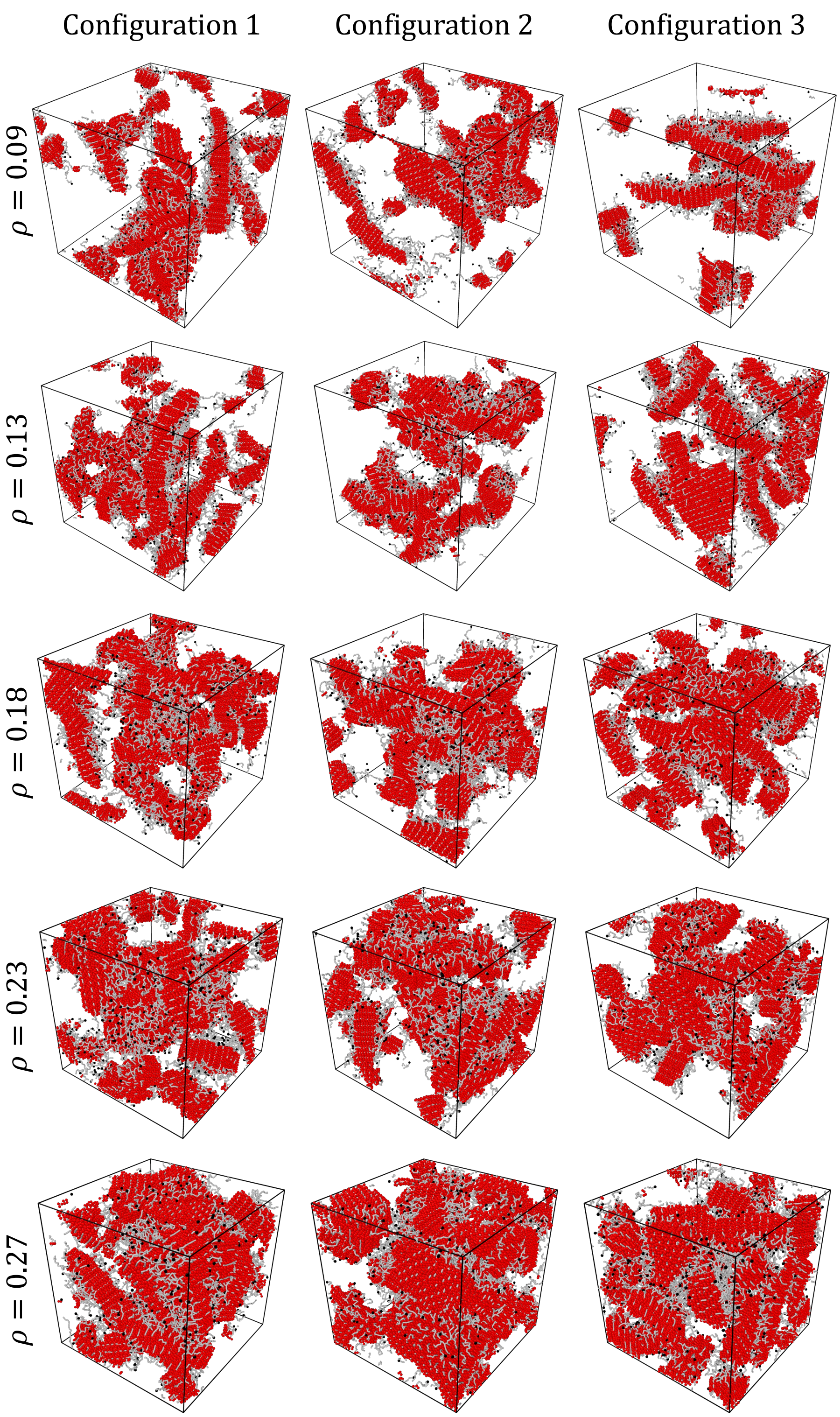

Figure S13: Equilibrated N60 systems crystallized under quiescent conditions

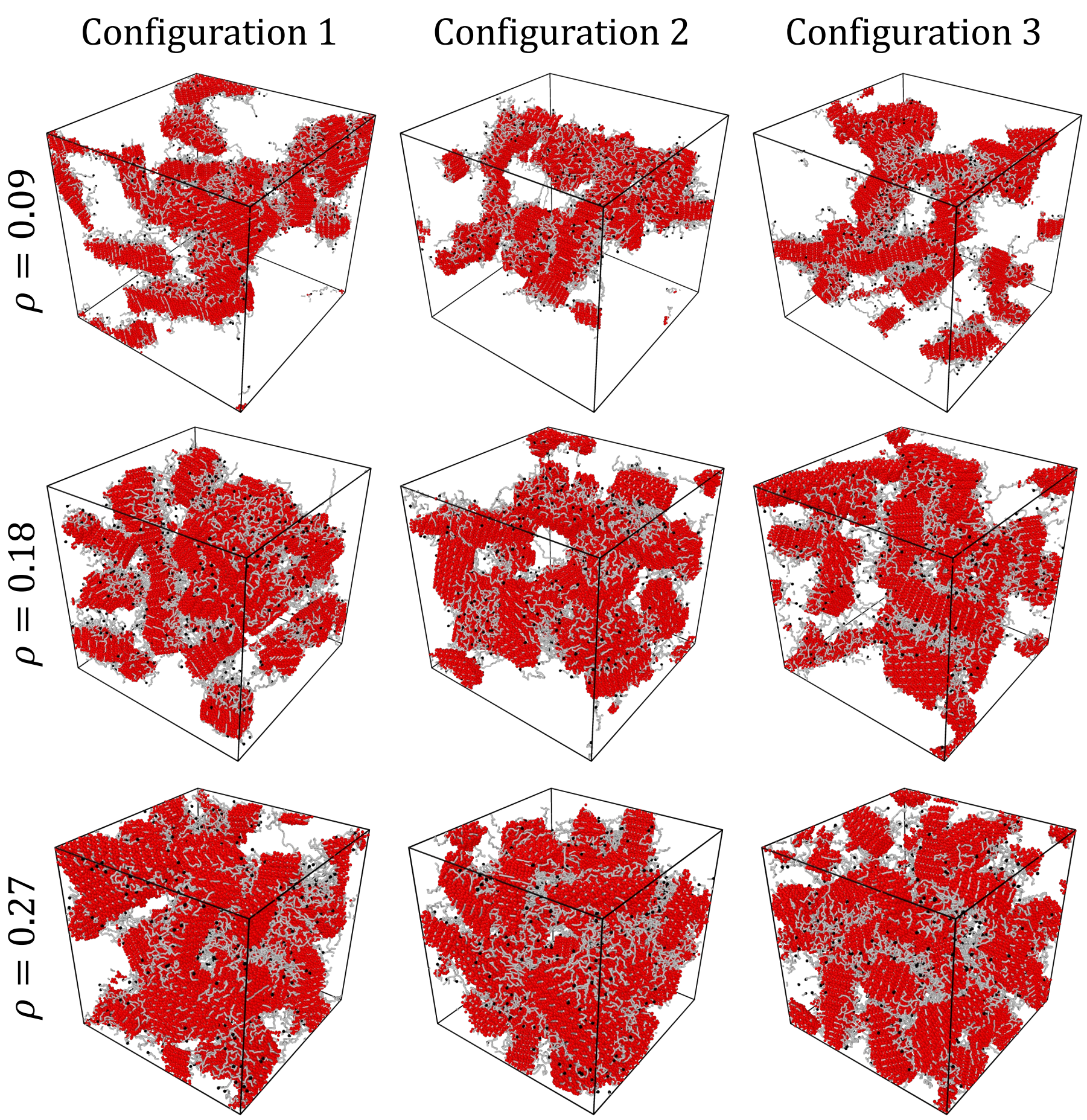

Figure S14: Equilibrated N80 systems crystallized under quiescent conditions

11 Sheared system snapshots and density profiles

Final snapshots and density profiles of each sheared system (Fig. S15-S18).

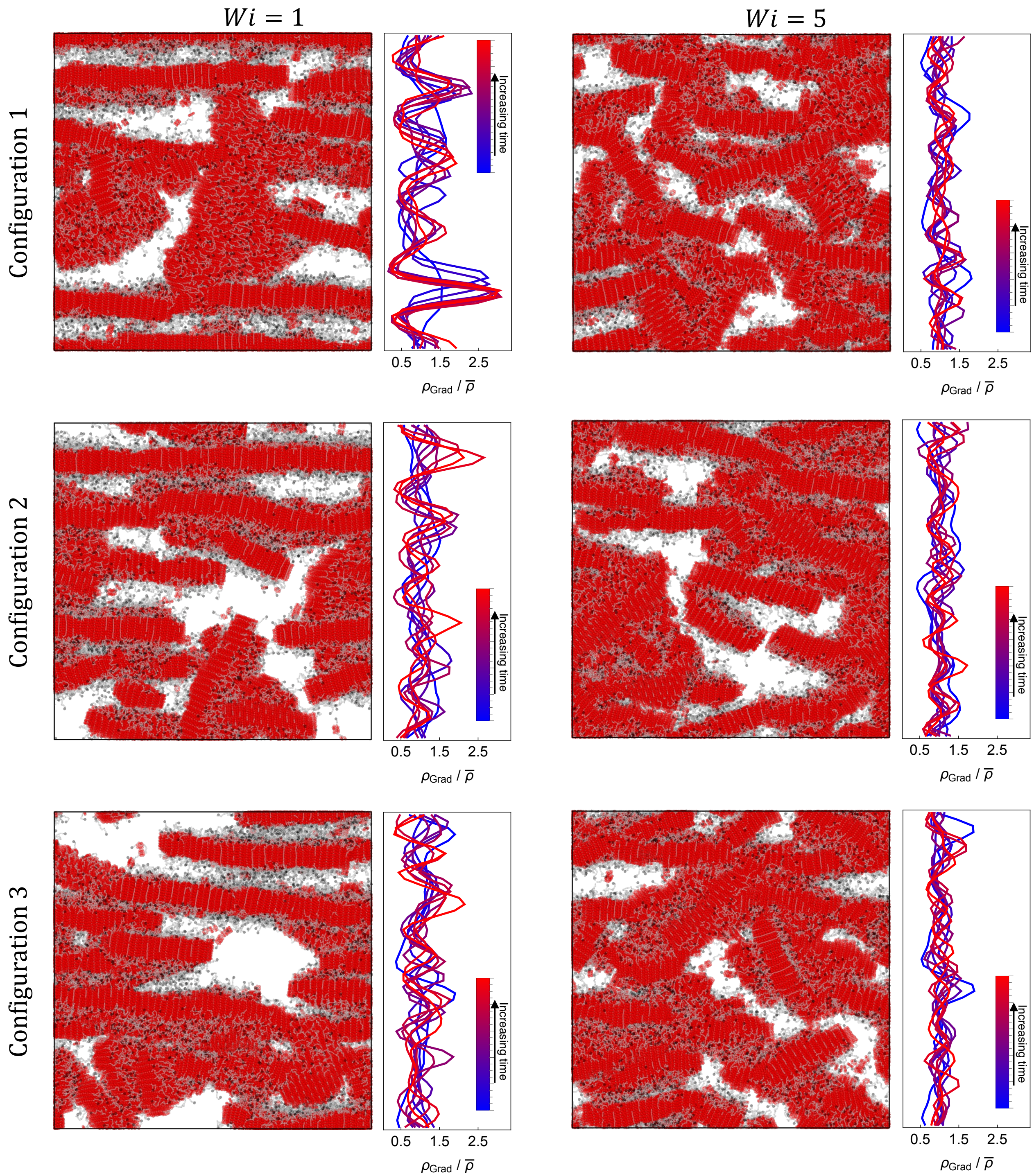

Figure S15: Time series density data for at $\rho \approx 0.18$

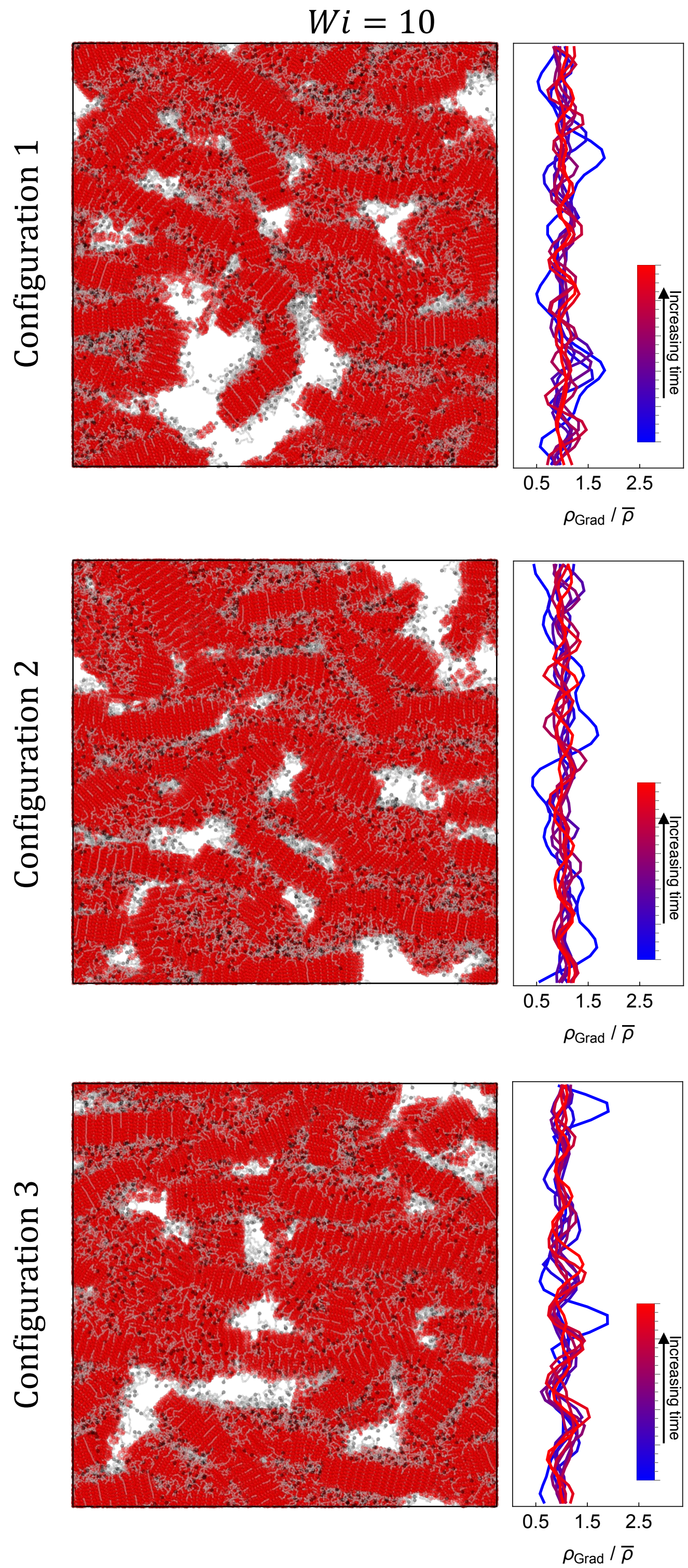

Figure S16: Time series density data for at $\rho \approx 0.18$

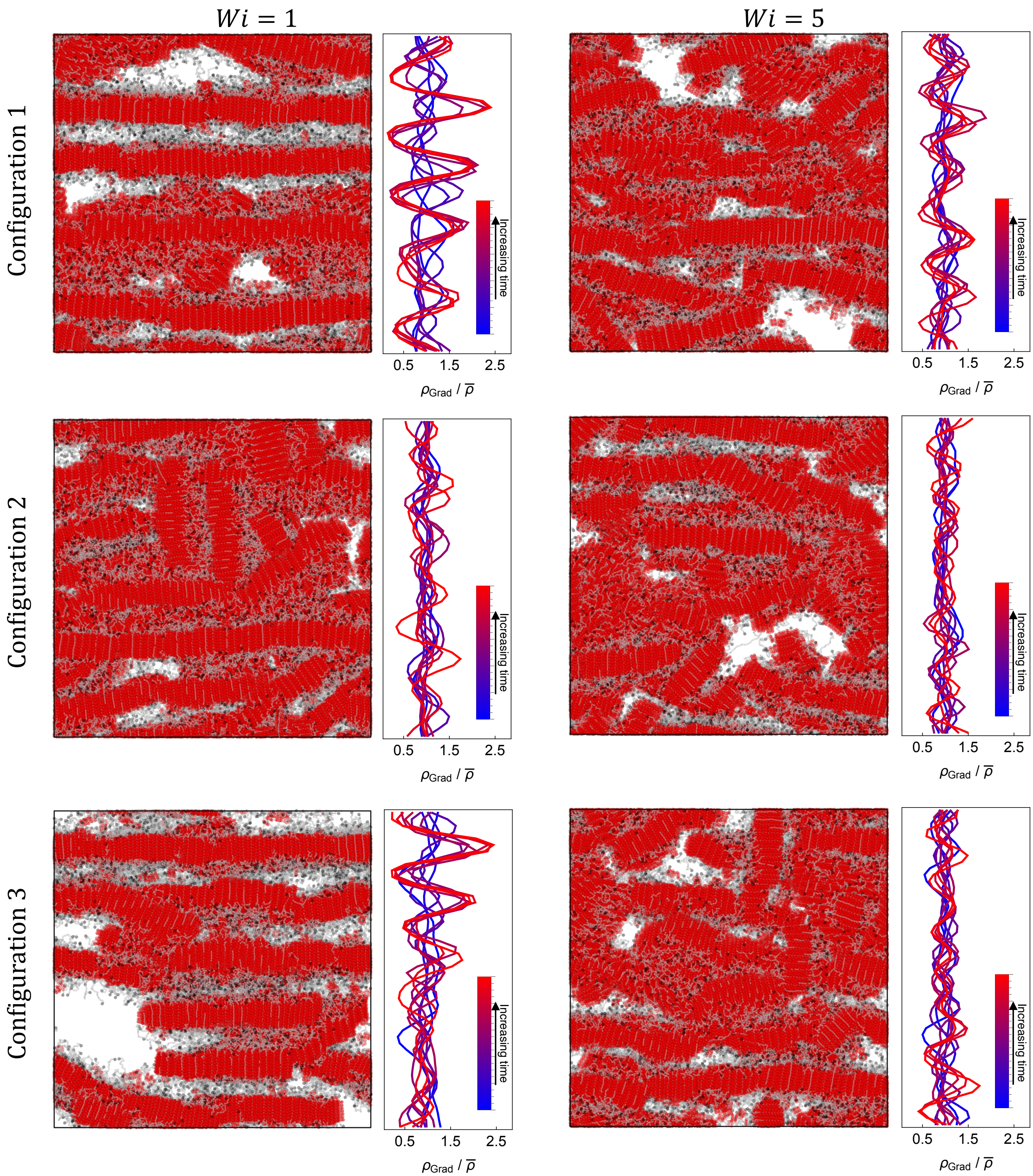

Figure S17: Time series density data for at $\rho \approx 0.27$

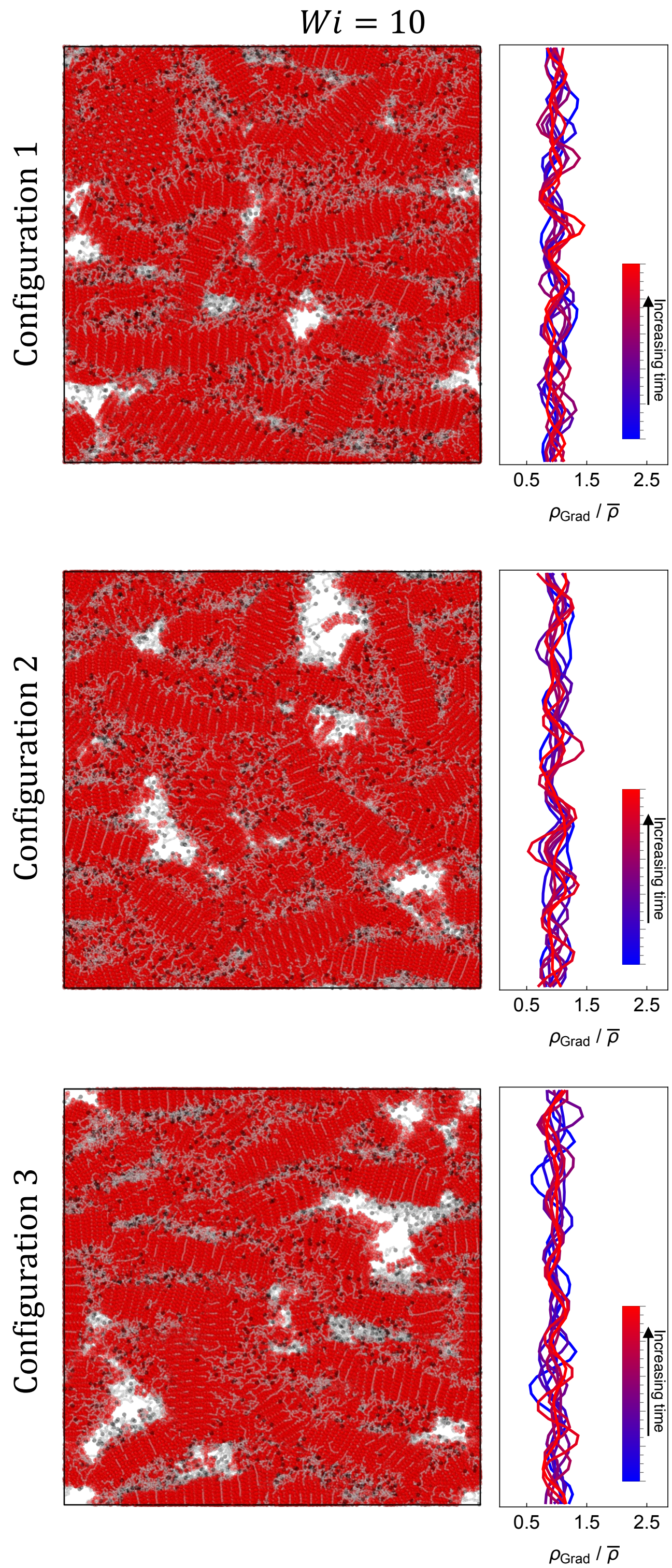

Figure S18: Time series density data for at $\rho \approx 0.27$

12 Re-equilibrated system snapshots

Final snapshots of each re-crystallized system (Fig. S19-S22).

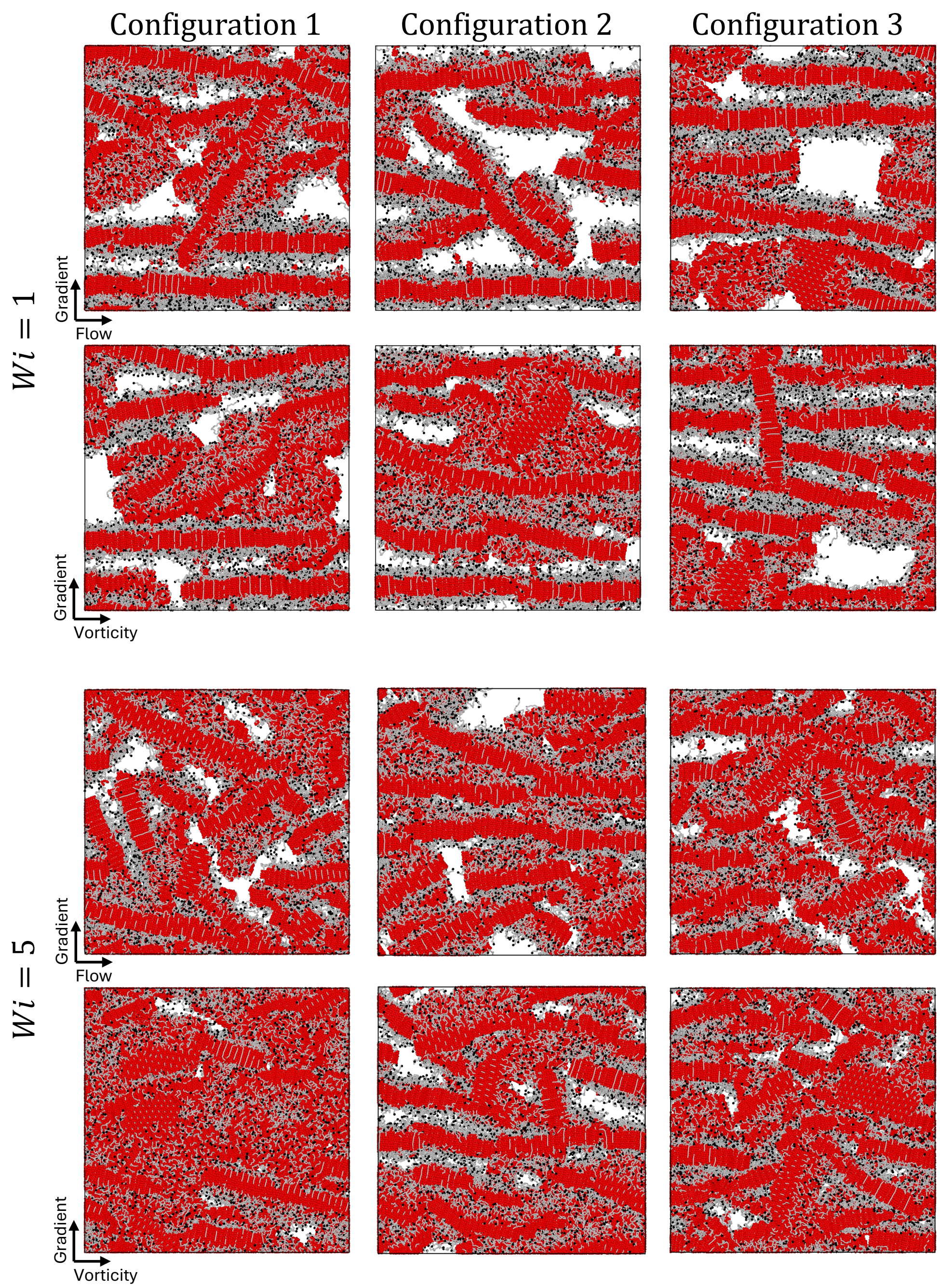

Figure S19: Re-equilibrated N40 systems at $\rho \approx 0.18$ re-crystallized under quiescent conditions

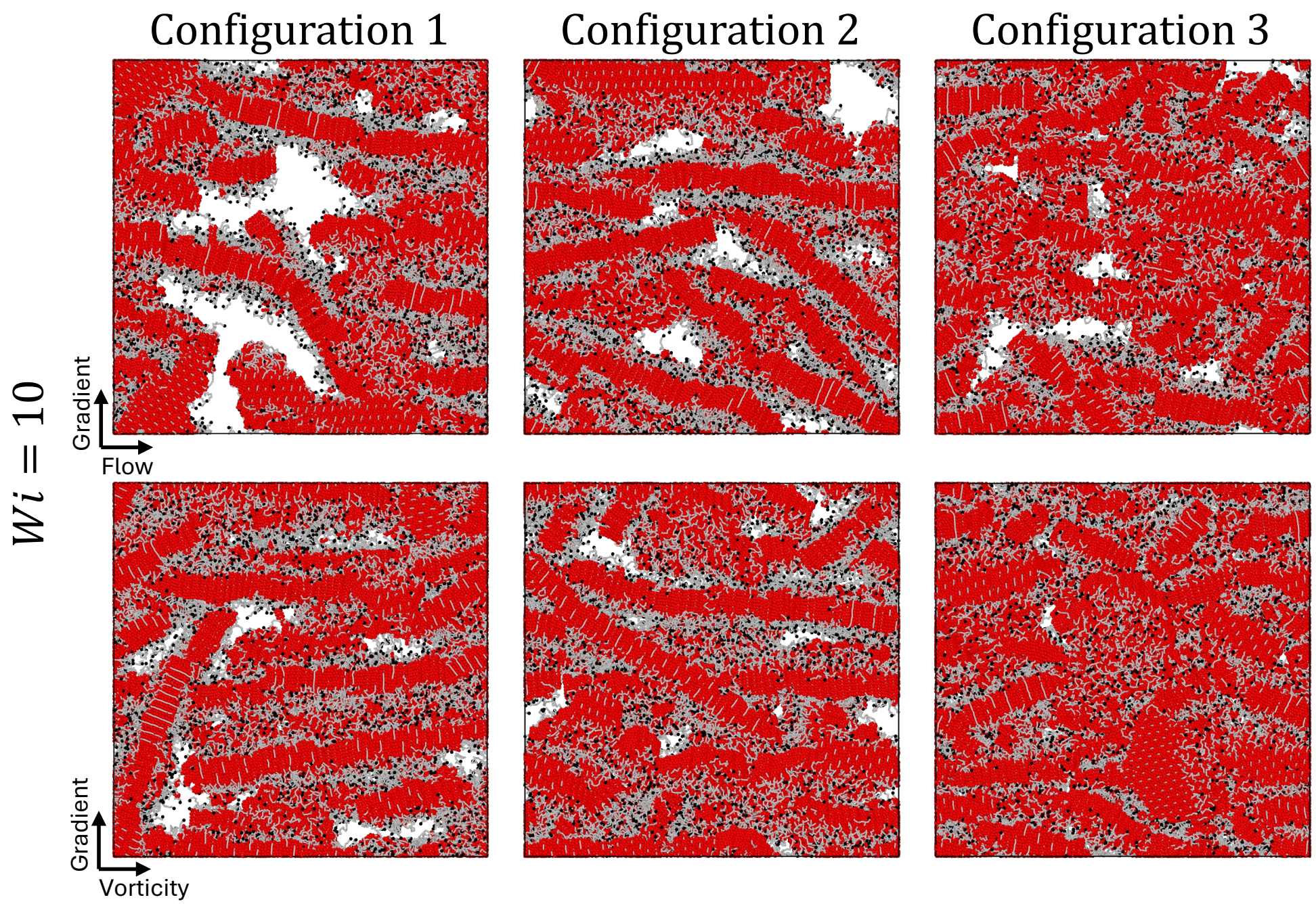

Figure S20: Re-equilibrated N40 systems at $\rho \approx 0.18$ re-crystallized under quiescent conditions

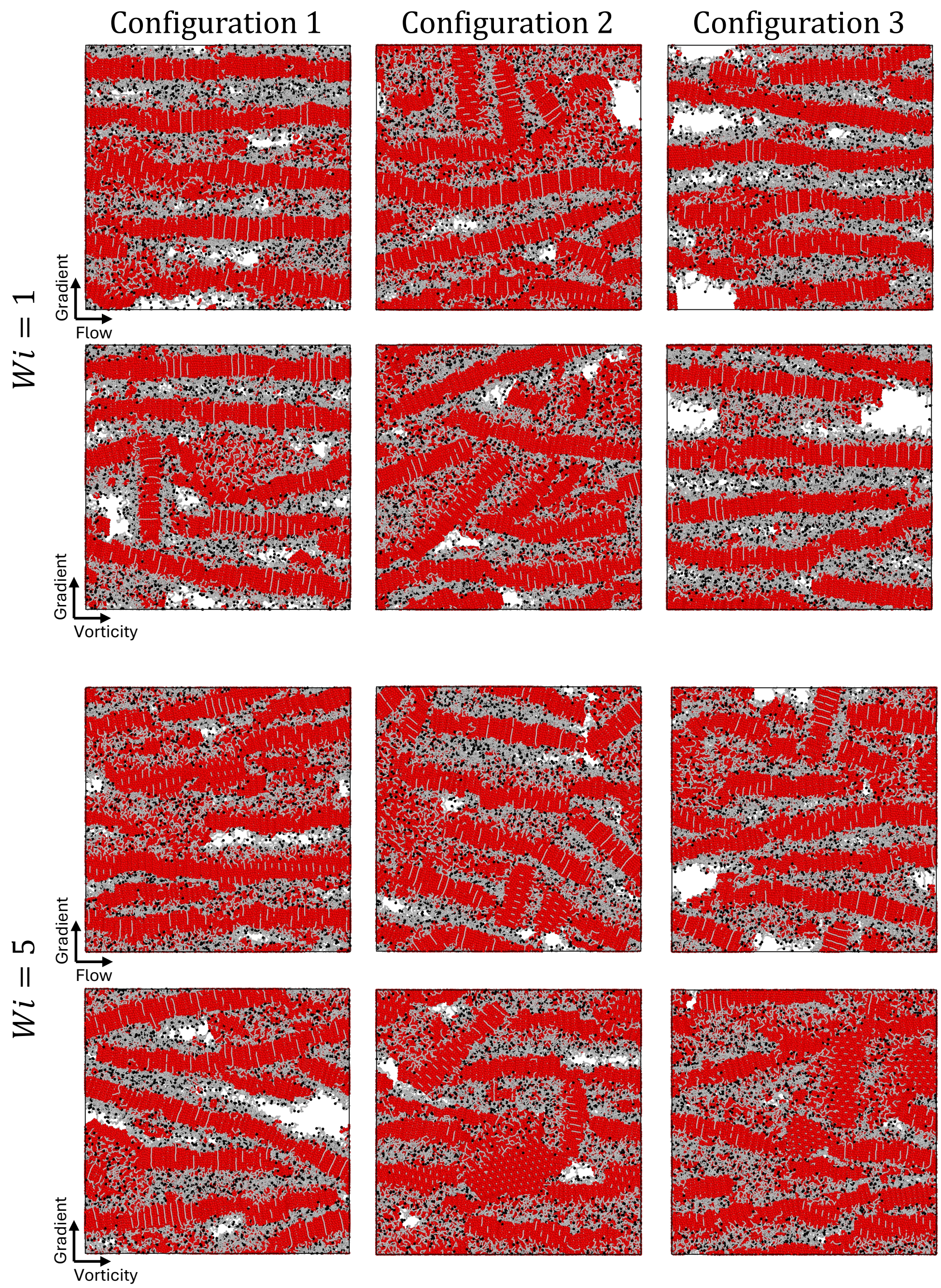

Figure S21: Re-equilibrated N40 systems at $\rho \approx 0.27$ re-crystallized under quiescent conditions

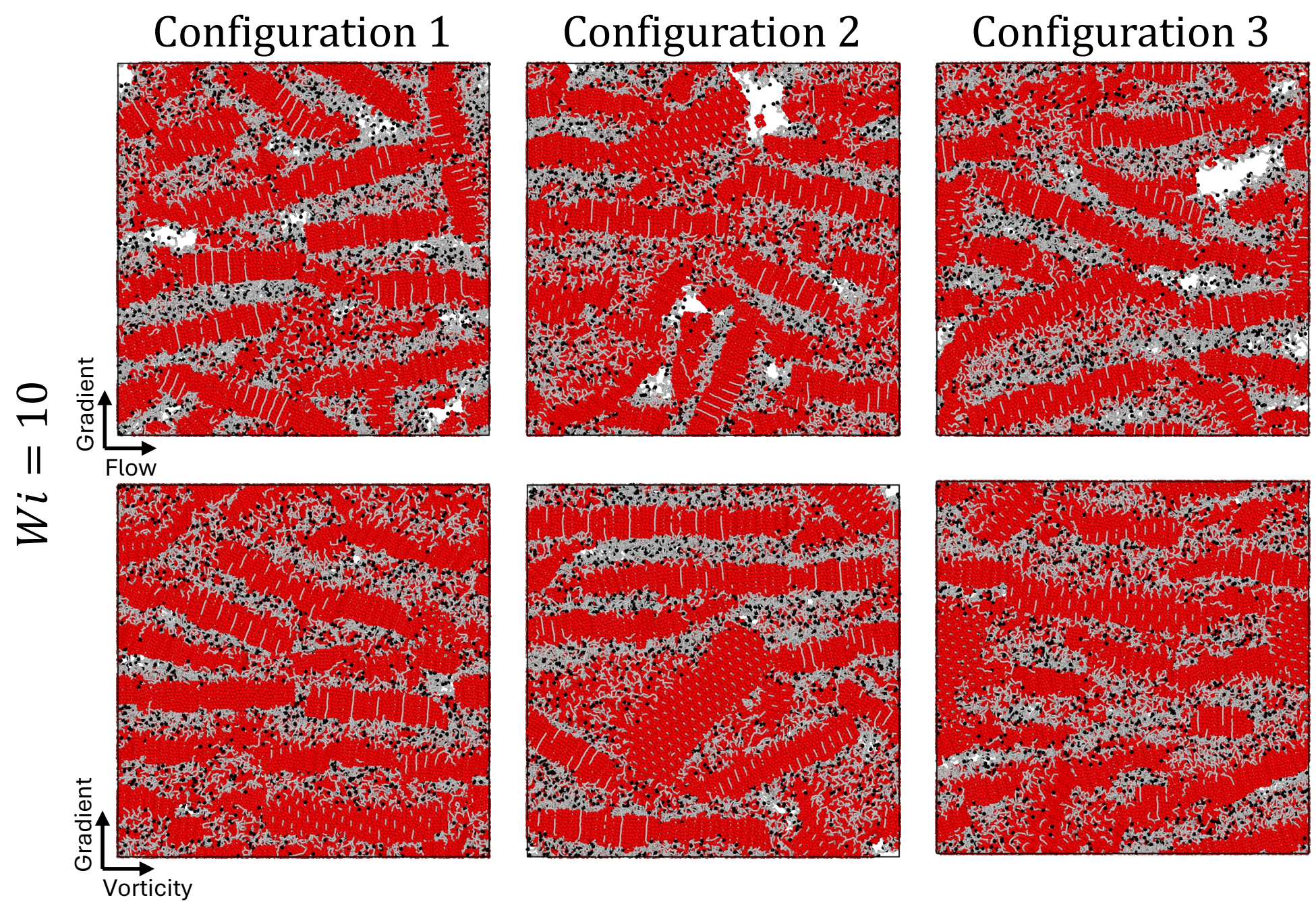

Figure S22: Re-equilibrated N40 systems at $\rho \approx 0.27$ re-crystallized under quiescent conditions

References

(1) Smith, C. D.; Ke, C.; Zhang, W. A multi-scale framework for predicting α-cyclodextrin assembly on polyethylene glycol axles. *Soft Matter* **2024**, *20*, 9068–9082, DOI: 10.1039/d4sm01048e.

(2) Uenuma, S.; Maeda, R.; Yokoyama, H.; Ito, K. Formation of isolated pseudo-polyrotaxane nanosheet consisting of α-cyclodextrin and poly(ethylene glycol). *Macromolecules* **2019**, *52*, 3881–3887, DOI: 10.1021/acs.macromol.9b00491.

(3) Zhang, M.; Liu, W.; Lin, Q.; Ke, C. Hierarchically templated synthesis of 3D-printed crosslinked cyclodextrins for lycopene harvesting. *Small* **2023**, *19*, 2300323, DOI: 10.1002/smll.202300323.

(4) Szejtli, J. Introduction and general overview of cyclodextrin chemistry. *Chemical Reviews* **1998**, *98*, 1743–1753.

(5) Malevanets, A.; Kapral, R. Mesoscopic model for solvent dynamics. *Journal of Chemical Physics* **1999**, *110*, 8605–8613, DOI: 10.1063/1.478857.

(6) Gompper, G.; Ihle, T.; Kroll, D. M.; Winkler, R. G. In *Advances in Polymer Science*, 1st ed.; Holm, C., Kremer, K., Eds.; Springer: Berlin, 2009; Vol. 221; pp 1–88, DOI: 10.1007/978-3-540-87706-6_1.

(7) Howard, M. P.; Panagiotopoulos, A. Z.; Nikoubashman, A. Efficient mesoscale hydrodynamics: Multiparticle collision dynamics with massively parallel GPU acceleration. *Computer Physics Communications* **2018**, *230*, 10–20, DOI: 10.1016/j.cpc.2018.04.009.

(8) Anderson, J. A.; Glaser, J.; Glotzer, S. C. HOOMD-blue: A Python package for high-performance molecular dynamics and hard particle Monte Carlo simulations. *Computational Materials Science* **2020**, *173*, DOI: 10.1016/j.commatsci.2019.109363.

(9) Noguchi, H.; Kikuchi, N.; Gompper, G. Particle-based mesoscale hydrodynamic techniques. *Europhysics Letters* **2007**, *78*, 10005, DOI: 10.1209/0295-5075/78/10005.

(10) Nikoubashman, A.; Howard, M. P. Equilibrium dynamics and shear rheology of semiflexible polymers in solution. *Macromolecules* **2017**, *50*, 8279–8289, DOI: 10.1021/acs.macromol.7b01876.

(11) Ripoll, M.; Mussawisade, K.; Winkler, R. G.; Gompper, G. Low-Reynolds-number hydrodynamics of complex fluids by multi-particle- collision dynamics. *Europhysics Letters* **2004**, *68*, 106–112, DOI: 10.1209/epl/i2003-10310-1.

(12) Noguchi, H.; Gompper, G. Transport coefficients of off-lattice mesoscale-hydrodynamics

simulation techniques. *Physical Review E - Statistical, Nonlinear, and Soft Matter Physics* **2008**, *78*, 016706, DOI: 10.1103/PhysRevE.78.016706.

(13) Müller-Plathe, F. Reversing the perturbation in nonequilibrium molecular dynamics: An easy way to calculate the shear viscosity of fluids. *Physical Review E* **1999**, *59*, 4894–4898.

(14) Tenney, C. M.; Maginn, E. J. Limitations and recommendations for the calculation of shear viscosity using reverse nonequilibrium molecular dynamics. *Journal of Chemical Physics* **2010**, *132*, 014103, DOI: 10.1063/1.3276454.

(15) Ihle, T.; Kroll, D. M. Stochastic rotation dynamics: A galilean-invariant mesoscopic model for fluid flow. *Physical Review E* **2001**, *63*, 020201(R), DOI: 10.1103/PhysRevE.63.020201.
